# Bridging the Scale Gap: A Multi-Scale Feature Enhancement Framework for UAV Aerial Image Object Detection

**DOI:** 10.3390/s26185832

**Published:** 2026-09-14

**Authors:** Dan Shan, Zanqi Qiu, Dadi Cai, Carlos Ferran, Xuan Tong, Dongming Liu

**Affiliations:** 1 School of Electrical and Control Engineering, Shenyang Jianzhu University, Shenyang 110168, China; shandan0405@sjzu.edu.cn (D.S.); qlwgfff@stu.sjzu.edu.cn (Z.Q.); ddcai0914@stu.sjzu.edu.cn (D.C.); 1762509855@stu.sjzu.edu.cn (X.T.); 2 College of Business, Governors State University, University Park, IL 60484, USA; cferran@govst.edu

**Keywords:** enhanced feature connection module, feature channel attention, Reinforced Attention Feedback, unified query supervision loss

## Abstract

**Highlights:**

**What are the main findings?**

- A UAV-specific RT-DETR framework, termed MSF-DETR, is developed to improve feature representation and query optimization for aerial object detection.
- Experiments on the DIOR and DOTA datasets show that MSF-DETR consistently outperforms RT-DETR and representative baseline detectors, achieving mAP50 scores of 86.3% and 77.2%, respectively.

**What are the implications of the main findings?**

- The results demonstrate that coordinated cross-scale feature enhancement, channel discrimination, spatial-detail preservation, and query supervision are effective for challenging UAV scenes involving small and densely distributed targets.
- The proposed design provides an effective balance between detection performance and computational efficiency, indicating its potential for practical UAV remote-sensing applications.

**Abstract:**

Unmanned aerial vehicle (UAV) imagery is a core data source for remote sensing interpretation, intelligent transportation, urban monitoring, and disaster assessment, yet its large scale variation, dense object distributions, and complex backgrounds continue to challenge automated detection systems. Transformer-based detectors offer strong global modeling capacity, but existing implementations still suffer from insufficient multi-scale feature interaction, weak discriminative representation, and loss of fine-grained spatial detail, which together limit performance on small and densely arranged targets. This paper proposes MSF-DETR, a multi-scale feature enhancement framework built on RT-DETR that integrates four coordinated components: an Enhanced Feature Connection (EFC) module for adaptive cross-scale interaction, a Feature Channel Attention (FCA) module for frequency-domain discriminative enhancement, a Reinforced Attention Feedback Module (RAFM) for spatial-detail preservation within the Transformer encoder, and a Unified Query Supervision Loss (UQSL) for stable dense-scene supervision. On the DIOR benchmark, MSF-DETR achieves 86.3% mAP50 and 64.4% mAP50–95, improving on the RT-DETR baseline by 2.8 and 2.6 percentage points, respectively; on DOTA, it reaches 77.2% mAP50 and 48.8% mAP50–95, improvements of 4.7 and 4.6 points. These results demonstrate that jointly coordinating multi-scale fusion, channel discrimination, spatial-detail retention, and query-level supervision, rather than stacking independent modules, yields measurable robustness gains for small and densely distributed objects in UAV aerial imagery.

## 1. Introduction

Unmanned aerial vehicle (UAV) imagery has become an important visual data source for remote sensing interpretation, intelligent transportation, urban monitoring, disaster assessment, and military reconnaissance. Compared with natural-scene images, UAV aerial images usually present a more challenging visual distribution: objects are often observed from a top-down or oblique perspective, their apparent scales change sharply with flight altitude, and small targets are frequently embedded in complex backgrounds such as buildings, roads, vegetation, shadows, and water surfaces. In addition, the dense spatial arrangement of objects further increases inter-instance ambiguity, making it difficult for detection models to maintain both accurate localization and reliable classification. These characteristics indicate that UAV object detection is not merely a standard object detection problem under a new data source, but a more fundamental problem of robust visual representation under scale-density-background coupling.

Existing object detectors have made substantial progress in addressing part of this problem. Two-stage detectors usually achieve strong localization accuracy through region proposal generation [1,2], but their cascaded inference pipelines limit real-time deployment in UAV scenarios. One-stage detectors, represented by the YOLO series [3], provide high inference efficiency and have been widely used in practical applications; however, their detection performance tends to degrade when targets are extremely small, densely distributed, or visually confused with background structures. Transformer-based detectors reformulate object detection as a set prediction task and introduce global attention mechanisms, which provide a promising paradigm for modeling long-range dependencies and eliminating hand-designed post-processing procedures. Nevertheless, when directly applied to UAV aerial imagery, Transformer-based detectors still face a critical limitation: global modeling alone cannot sufficiently resolve the loss of small-object details, the semantic inconsistency among multi-scale features, and the unstable query-to-object assignment in dense scenes.

Therefore, the central theoretical issue addressed in this paper is how to construct an end-to-end detection framework in which feature representation and query optimization are jointly coordinated for UAV aerial object detection. More specifically, an effective detector for UAV imagery should satisfy three requirements. First, it should reduce the semantic gap between shallow spatial features and deep semantic features, because small objects rely heavily on fine-grained local details while classification requires high-level semantic abstraction. Second, it should enhance discriminative feature responses under complex backgrounds, since redundant background regions may occupy a large proportion of aerial images and weaken the representation of small targets. Third, it should preserve spatial details during Transformer encoding and provide stable supervision for object queries, because dense object distributions often lead to ambiguous matching and insufficient positive supervision. These requirements suggest that the key to improving UAV object detection lies not in simply stacking attention or fusion modules, but in establishing a unified representation–optimization mechanism that simultaneously strengthens multi-scale feature interaction, channel-wise discrimination, spatial-detail preservation, and query-level learning stability.

To address this issue, this paper proposes MSF-DETR, a multi-scale feature enhancement-based RT-DETR framework for UAV aerial object detection. The method is built upon RT-DETR and introduces four coordinated components rather than independent architectural modifications. First, an Enhanced Feature Connection module is designed to adaptively reconstruct and fuse hierarchical features, thereby improving semantic consistency across scales and enhancing the representation of small and densely distributed objects. Second, a Feature Channel Attention module is introduced to incorporate frequency-domain modeling and channel-wise recalibration, enabling the network to emphasize task-relevant feature responses while suppressing background redundancy. Third, a Reinforced Attention Feedback Module is embedded into the Transformer encoder to recycle and refine intermediate attention features, which helps preserve fine-grained spatial information during deep encoding. Finally, a Unified Query Supervision Loss is developed to integrate Hungarian matching, denoising supervision, and one-to-many assignment into a coordinated optimization framework, improving query learning stability in dense detection scenarios. These designs correspond to the limitations identified in the current manuscript, where RT-DETR is noted to have limited multi-scale interaction and restricted performance for small objects and complex backgrounds, while the proposed method strengthens feature interaction, channel representation, attention learning, and query optimization.

The contributions of this work are summarized as follows:

1. A unified representation–optimization perspective is introduced for UAV aerial object detection. Instead of treating scale variation, background interference, spatial-detail degradation, and query instability as isolated problems, this work formulates them as coupled representation and optimization challenges in Transformer-based detection.
2. An Enhanced Feature Connection module is proposed for adaptive cross-scale feature reconstruction. By dynamically modeling interactions between shallow spatial details and deep semantic features, the module improves multi-scale semantic consistency and strengthens small-object representation.
3. A Feature Channel Attention module is developed to enhance discriminative feature learning. By combining frequency-domain modeling with channel-wise recalibration, the module suppresses redundant background responses and improves the signal-to-noise ratio of aerial object features.
4. A Reinforced Attention Feedback Module is embedded into the Transformer encoder. The module reintroduces refined intermediate attention features into subsequent encoding layers, reducing the loss of fine-grained spatial information and improving localization accuracy for small and occluded objects.
5. A Unified Query Supervision Loss is designed to stabilize dense-scene query optimization. By jointly coordinating one-to-one matching, contrastive denoising, and one-to-many assignment, the loss function provides richer and more stable supervision for object queries.

Extensive experiments are conducted on the DIOR and DOTA datasets to evaluate the effectiveness of the proposed framework. The experimental results show that MSF-DETR achieves 86.3% mAP50 and 64.4% mAP50–95 on DIOR, and 77.2% mAP50 and 48.8% mAP50–95 on DOTA, outperforming the RT-DETR baseline and several representative CNN-, YOLO-, and Transformer-based detectors. These results demonstrate that the proposed framework improves detection accuracy while maintaining a practical balance between performance and computational cost.

The remainder of this paper is organized as follows. Section 2 reviews related work on UAV aerial object detection and deep learning-based detection frameworks. Section 3 presents the proposed MSF-DETR framework and its key components. Section 4 reports the experimental settings, ablation studies, quantitative comparisons, and qualitative analysis. Section 5 discusses the findings, limitations, and future research directions.

## 2. Related Work

UAV aerial object detection has been extensively studied because aerial imagery presents substantial variations in object scale, spatial density, viewing perspective, and background complexity [4]. In particular, small objects may occupy only a limited number of pixels, while densely distributed instances and visually similar background structures make feature discrimination and localization more difficult [5]. Existing studies have explored multi-scale feature representation, attention-based enhancement, Transformer encoding, and query optimization to address these challenges. Rather than reviewing these methods only according to detector architecture, this section organizes existing research around four closely related technical issues: cross-level feature interaction, discriminative feature learning under complex backgrounds, spatial-detail preservation in Transformer encoding, and stable supervision of object queries.

### 2.1. Cross-Level Feature Interaction and Multi-Scale Representation

A fundamental challenge in UAV aerial object detection is the semantic gap between shallow spatial features and deep semantic features. Shallow layers retain relatively precise positional and boundary information, which is important for small objects, whereas deep layers contain stronger semantic representations but lose fine spatial details due to successive downsampling. When these heterogeneous features are insufficiently coordinated, the resulting representation may be semantically inconsistent across scales, leading to degraded detection performance for small and densely distributed objects.

Multi-scale feature fusion has therefore become an important research direction. Liang et al. proposed a small-object detection framework for UAV images that combines feature fusion, scaling-based single-shot detection, and spatial context analysis. Their results demonstrate that integrating information from different feature levels can substantially improve the representation of small aerial objects. EfficientDet [6] further introduced a weighted bidirectional feature pyramid network to perform repeated bidirectional information exchange across feature levels, providing an efficient solution for multi-scale object representation.

Recent UAV-oriented detectors have continued to improve cross-level feature interaction. YOLO-MARS [7] enhances YOLOv8n through a multi-level attention residual mechanism and an improved feature fusion structure to strengthen the representation of small aerial objects. Xu et al. [8] further investigated small-object detection based on YOLOv8n for UAV images, highlighting the importance of preserving fine-scale information. HierLight-YOLO [9] employs hierarchical feature aggregation and lightweight operations to improve feature representation while controlling computational complexity. MASF-YOLO [10] incorporates multi-scale feature processing and attention mechanisms into YOLOv11 to enhance small-object detection from drone views. YOLO-Drone [11] similarly redesigns the YOLOv8 architecture to improve the representation of tiny objects in UAV imagery.

Although these methods demonstrate the effectiveness of multi-scale feature aggregation, most of them focus on increasing the amount of information exchanged between feature levels rather than explicitly improving the semantic consistency between shallow spatial information and deep semantic representations. Moreover, increasingly complex fusion structures may introduce additional computational costs. These observations indicate that effective cross-scale feature interaction should reconstruct complementary information between different levels rather than simply concatenate or aggregate them.

### 2.2. Discriminative Feature Learning Under Complex Backgrounds

Feature representation alone is insufficient when target objects are embedded in highly complex backgrounds. UAV images frequently contain buildings, roads, vegetation, shadows, water surfaces, clouds, and other structures that may generate strong visual responses unrelated to the target. When background regions occupy a large proportion of an aerial image, these redundant responses can weaken target-related features and reduce the discriminability of object representations.

Attention mechanisms have been widely explored to address this problem by assigning larger weights to informative features. YOLO-MARS, for example, introduces an attention-based residual mechanism to emphasize useful target information. MASF-YOLO further integrates attention operations with multi-scale feature aggregation to improve the representation of small aerial targets. The feature enhancement strategies used in YOLO-Drone also demonstrate the importance of selectively strengthening target-related information in aerial scenes.

Contextual modeling provides another complementary approach. Liang et al. incorporated spatial context analysis into small-object detection, demonstrating that surrounding information can provide useful clues when local appearance is insufficient for reliable classification. EfficientDet improves multi-scale contextual representation through bidirectional feature aggregation, while subsequent UAV-oriented methods exploit hierarchical or multi-scale representations to incorporate broader contextual information.

However, existing attention and context-based approaches generally emphasize informative responses without explicitly considering the frequency characteristics and channel-wise redundancy of aerial features. As a result, visually strong but semantically irrelevant background patterns may still be propagated through the network. This limitation becomes especially important in dense aerial scenes, where target-background ambiguity is high. Therefore, a more discriminative representation mechanism should strengthen task-relevant feature channels while simultaneously suppressing redundant background responses.

### 2.3. Spatial-Detail Preservation in Transformer Encoding

Transformer-based architectures have introduced global dependency modeling into visual recognition and have demonstrated strong capability in capturing long-range relationships [12]. DETR [13] formulated object detection as a direct set prediction problem and replaced conventional hand-designed components, such as anchor generation and non-maximum suppression, with a Transformer encoder–decoder architecture and bipartite matching. This formulation established a unified end-to-end paradigm for object detection.

Despite these advantages, the original DETR exhibits slow convergence and limited sensitivity to small objects, partly because global attention does not explicitly emphasize fine-grained spatial structures. Deformable DETR [14] alleviates this limitation by restricting attention to a sparse set of informative sampling locations, improving convergence efficiency and small-object representation. These developments indicate that efficient spatial interaction is critical when Transformer architectures are applied to detection tasks involving objects with limited spatial extent.

Vision Transformer research has further demonstrated the effectiveness of hierarchical and multi-scale visual representations for improving spatial feature modeling [12]. Nevertheless, Transformer encoding still presents a potential trade-off between global semantic abstraction and local spatial preservation. As features are progressively encoded, fine boundaries, local textures, and weak spatial responses may be diluted by deeper semantic interactions. For aerial images containing small and densely distributed objects, this degradation may directly affect localization accuracy.

Existing approaches mainly address this issue by modifying the attention operation or introducing multi-scale feature structures. However, these strategies do not necessarily make effective use of intermediate attention information that may contain valuable fine-grained spatial responses. This suggests that preserving spatial details during Transformer encoding requires not only improved attention sampling but also mechanisms capable of reusing and refining informative intermediate representations.

### 2.4. Object Query Learning and Stable Prediction Supervision

Another important characteristic of DETR-style detectors is their reliance on object queries. Instead of generating dense candidate predictions as in conventional one-stage detectors, DETR uses a fixed set of learnable object queries to represent potential object instances and employs bipartite matching to establish one-to-one correspondence between predictions and ground-truth objects [13]. This formulation simplifies the detection pipeline but makes query optimization an important factor affecting convergence and final detection performance.

The original DETR requires a relatively long training process before object queries can learn meaningful object representations [13]. Deformable DETR improves query-feature interaction by introducing reference points and deformable attention, enabling queries to focus on informative local regions [14]. These improvements demonstrate that the quality of spatial features supplied to the Transformer decoder and the manner in which queries interact with those features are closely related to detection stability.

The difficulty becomes more pronounced in dense aerial scenes. When many objects appear simultaneously, one-to-one Hungarian matching provides only limited positive supervision to individual queries. Queries that are spatially close to the same target may therefore receive ambiguous optimization signals, while potential positive predictions may be discarded because only one prediction is selected for a ground-truth object. Denoising-based training and auxiliary query supervision have consequently become important strategies for improving the optimization of DETR-like detectors.

Nevertheless, improving query learning through a single supervision mechanism does not completely solve the problem. Stable query optimization depends on the consistency of feature representations, the quality of matching, and the availability of sufficiently informative positive signals. Therefore, a more effective supervision strategy should coordinate one-to-one matching with richer query-level supervision so that object queries can receive stable optimization signals without sacrificing the advantages of end-to-end set prediction.

### 2.5. Summary and Research Gap

The existing literature provides complementary solutions to the major challenges of UAV aerial object detection. Multi-scale feature fusion and hierarchical architectures improve the interaction between low- and high-level representations, while attention and contextual modeling enhance feature discrimination in complex scenes. Transformer-based methods provide powerful global dependency modeling, but preserving fine spatial details during encoding remains challenging. Furthermore, DETR-style query-based prediction introduces an additional optimization issue because object queries depend strongly on the quality of encoded features and the stability of their supervision.

These studies collectively suggest that the four issues identified in the Introduction are strongly coupled. Inadequate cross-scale interaction may weaken small-object representation; excessive background responses may reduce feature discriminability; the loss of intermediate spatial information may further impair precise localization; and insufficient or unstable query supervision may prevent the detector from fully exploiting the available feature representations. Therefore, simply stacking independent attention, fusion, or Transformer modules is unlikely to provide a unified solution.

Motivated by these observations, this paper proposes MSF-DETR, which addresses the above challenges through coordinated representation enhancement and query optimization. The Enhanced Feature Connection module focuses on improving cross-level semantic consistency; the Feature Channel Attention module enhances discriminative feature responses and suppresses background redundancy; the Reinforced Attention Feedback Module preserves informative spatial details during Transformer encoding; and the Unified Query Supervision Loss provides richer and more stable supervision for object queries. Together, these components establish a unified representation–optimization framework for robust UAV aerial object detection.

## 3. Methodology

### 3.1. RT-DETR

**Figure 1:**
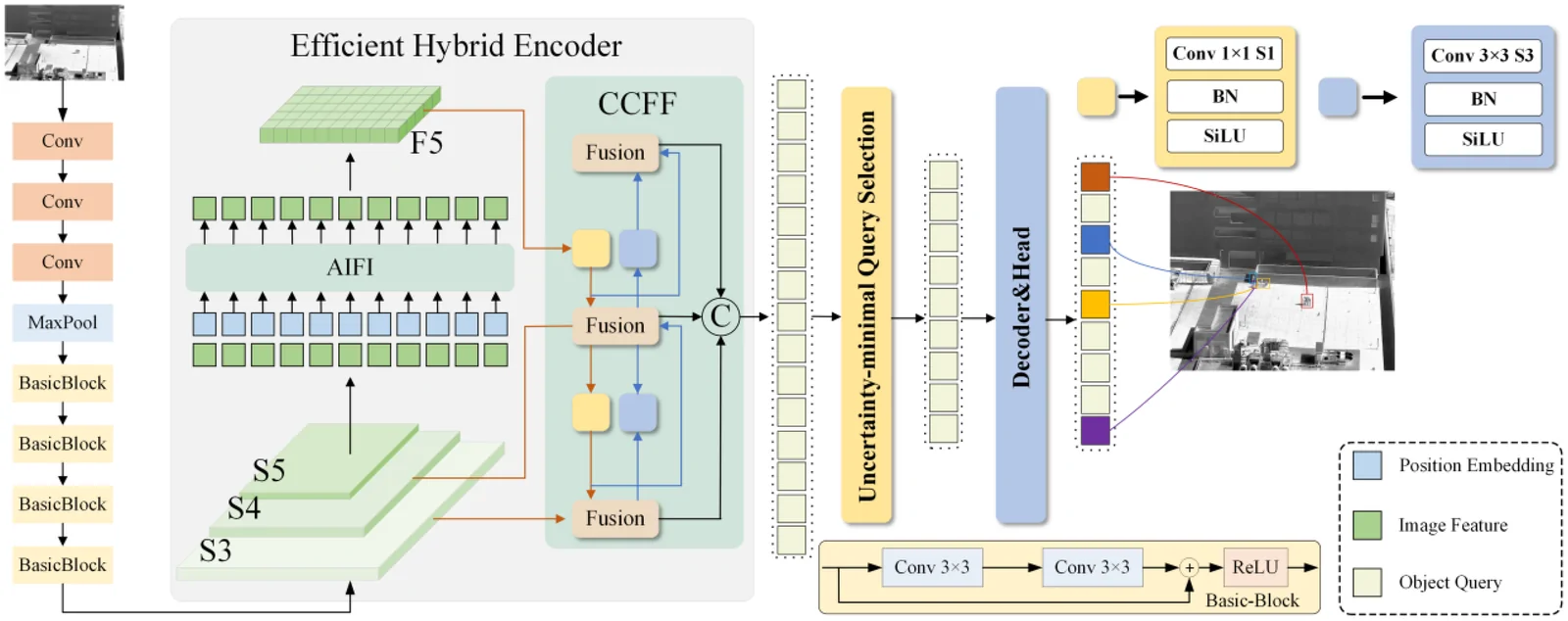
RT-DETR network structure.

RT-DETR is an end-to-end object detection framework that formulates detection as a direct set prediction problem using the Transformer architecture [15]. As illustrated in Figure 1, given an input image, multi-scale feature maps are first extracted by a convolutional backbone and then projected into a unified embedding space without relying on complex feature pyramid structures. The resulting features are flattened into a sequence and fed into a Transformer encoder based on multi-scale deformable attention, which enables efficient modeling of long-range dependencies by attending to a sparse set of sampling points across different feature levels.

The encoded features are subsequently processed by a Transformer decoder with a fixed number of learnable object queries, where each query predicts a class label and a bounding box. The final predictions are obtained via bipartite matching using the Hungarian algorithm, eliminating the need for post-processing steps such as non-maximum suppression. Although RT-DETR achieves a good balance between accuracy and efficiency, its simplified multi-scale processing and limited feature interaction may restrict its performance in challenging scenarios such as small object detection and complex backgrounds, which motivates the improvements proposed in this work.

### 3.2. MSF-DETR

**Figure 2:**
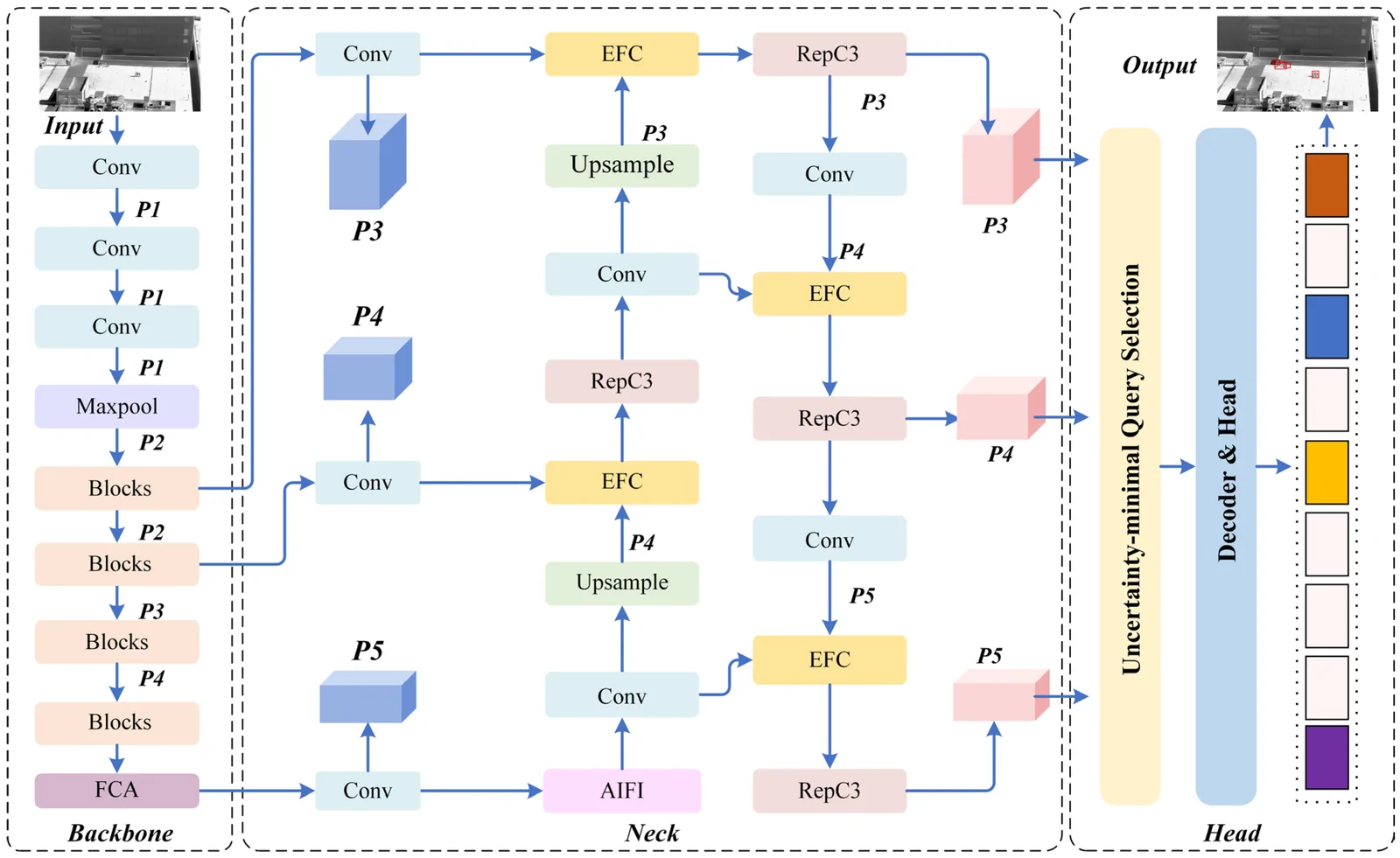
The architecture of the MSF-DETR. The red boxes in the output image indicate the detected object regions.

Building upon the RT-DETR framework, this paper proposes an enhanced detection model termed MSF-DETR, aiming to improve feature representation and detection robustness in UAV aerial imagery. As shown in Figure 2, the proposed method follows the standard RT-DETR pipeline while introducing several targeted enhancements.

EFC is introduced after the backbone to enable effective interaction among multi-scale features. The transformed features are further refined by an FCA module to enhance channel-wise discriminability. The enhanced representations are then fed into the Transformer encoder, where an RAFM is embedded to preserve and propagate fine-grained spatial information across layers. The decoder adopts the standard query-based detection mechanism to produce final predictions. In addition, a UQSL is designed to jointly optimize the training process, improving convergence stability and detection performance in challenging scenarios. Through these coordinated improvements, the proposed method achieves more discriminative feature learning and robust object detection.

### 3.3. Enhanced Feature Connection Module

UAV aerial images contain objects with substantial scale variations, and small targets often occupy only a few pixels. Shallow features preserve the spatial details required for their localization, whereas deep features provide stronger semantic information but suffer from reduced spatial resolution. This creates a semantic gap between shallow and deep features and makes direct feature fusion less effective for UAV target detection. Therefore, an adaptive cross-scale feature interaction mechanism is required to preserve fine-grained spatial details while incorporating high-level semantic information. Conventional element-wise addition assumes that features from different pyramid levels are already geometrically aligned and semantically compatible. However, in Transformer-based detectors, low-level features mainly encode spatial details whereas high-level features primarily contain semantic abstractions. Direct addition or concatenation therefore mixes heterogeneous feature distributions without explicitly reducing their semantic discrepancy, leading to information dilution and unstable feature propagation. EFC addresses this issue by dynamically reconstructing cross-scale features before fusion.

**Figure 3:**
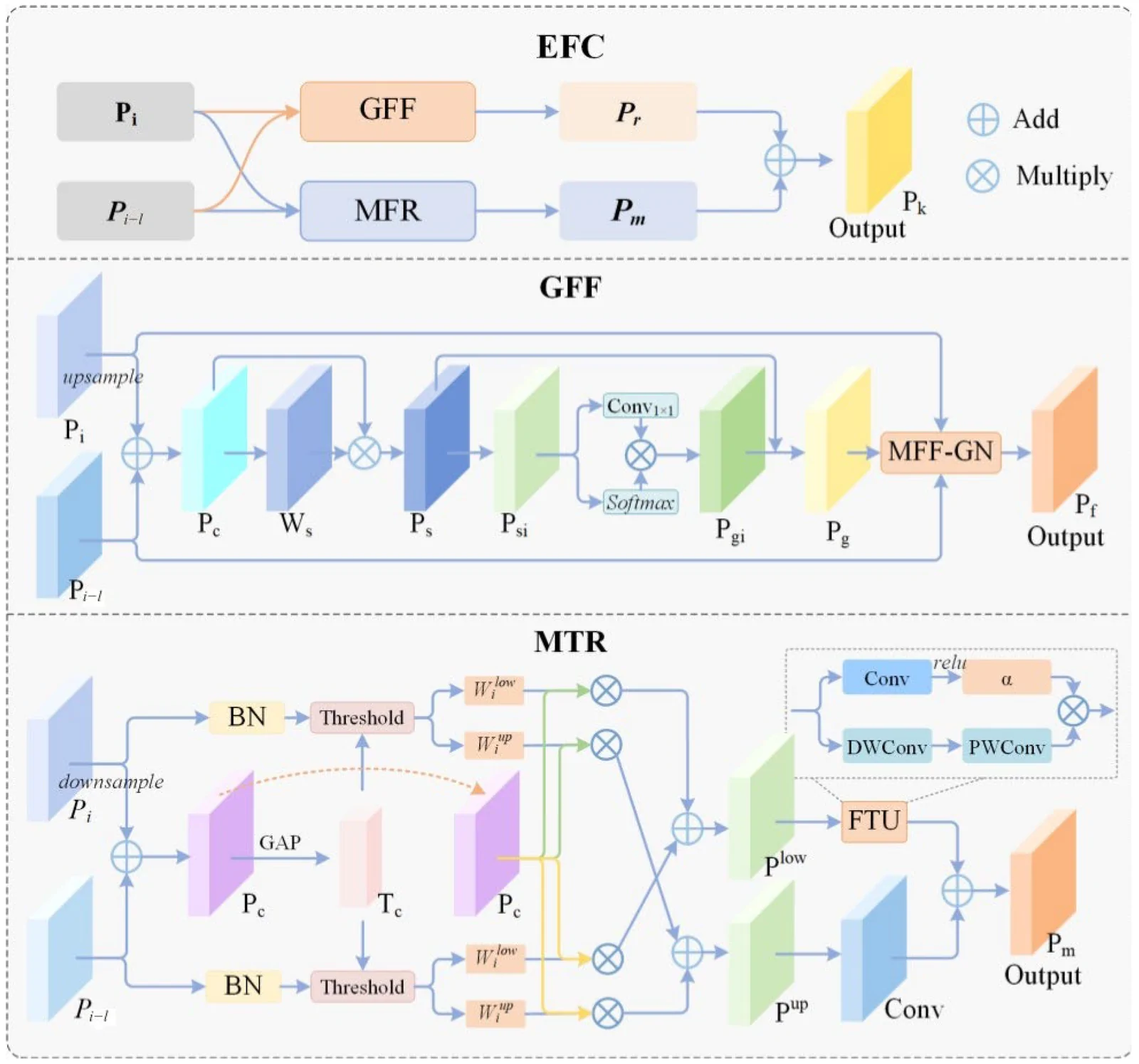
Schematic diagram of the enhanced feature connection module.

To address this UAV-specific challenge, the proposed Enhanced Feature Connection (EFC) module dynamically reconstructs and connects features from different hierarchical levels. By adaptively coordinating shallow spatial details and deep semantic information, EFC aims to obtain more consistent multi-scale representations and improve the detection of UAV targets with different apparent sizes. Traditional feature fusion methods typically rely on element-wise addition or channel concatenation. However, such strategies neglect the inherent differences between high-level semantic information and low-level spatial details, which may lead to insufficient semantic alignment and loss of fine-grained structures. The overall structure of the EFC module is shown in Figure 3.

To address this limitation, the proposed EFC module introduces a dynamic feature reconstruction mechanism, which enables adaptive interaction between deep and shallow features. The EFC module achieves dynamic interaction between deep semantic information and shallow spatial details by introducing Gated Feature Fusion (GFF) and Multi-scale Feature Reconstruction (MFR) mechanisms, thereby constructing a more stable and discriminative feature representation. Specifically, let the input features from adjacent levels be denoted as the deep feature $F_{d}\in R^{C\times H_{d}\times W_{d}}$, and the shallow feature $F_{s}\in R^{C\times H_{s}\times W_{s}}$. Since the spatial resolution of deep features is lower than that of shallow features, a spatial alignment operation is first performed:

(1) $${\overset{ˆ}{F}}_{d}=\varphi \left(U\left(F_{d}\right)\right),$$

where U(⋅) denotes the upsampling operation, and φ(⋅) represents a feature transformation implemented by a 1 × 1 convolution.

Then, the shallow feature and the aligned deep feature are jointly embedded to model cross-level correlations:

(2) $$F_{joint}=\varphi \left(\left[F_{s},{\overset{ˆ}{F}}_{d}\right]\right),$$

Based on the joint representation, a spatially adaptive gating map is generated:

(3) $$G=\sigma \left(\psi \left(F_{joint}\right)\right),$$

where ψ(⋅) denotes a convolutional mapping, and σ(⋅) is the Sigmoid activation function.

Subsequently, a dynamic feature reconstruction process is conducted to adaptively fuse the two features:

(4) $$F_{rec}=G\times F_{s}+\left(1-G\right)\times {\overset{ˆ}{F}}_{d},$$

To further enhance discriminative responses while suppressing background noise, a threshold-based activation function is introduced:

(5) $$F_{enh}=ReLU\left(F_{rec}-\tau \right),$$

where τ is initialized to 0.1 and updated through back-propagation together with the network parameters. The threshold τ controls the activation sensitivity of the gating mechanism. A relatively small value allows informative low-response regions to be preserved while effectively suppressing noisy feature responses. Empirical observations indicate that τ = 0.1 provides a favorable balance between feature preservation and background suppression across different datasets.

Finally, a residual connection is incorporated to preserve fine-grained spatial information:

(6) $$F_{out}=F_{enh}+F_{s},$$

Relationship to Prior Work. Unlike BiFPN, which performs repeated bidirectional weighted feature aggregation using fixed learnable fusion coefficients, the proposed EFC generates spatially adaptive gating maps conditioned on both deep semantic features and shallow spatial features. Moreover, unlike Attention Gate, which mainly suppresses irrelevant encoder responses during skip connections, EFC explicitly reconstructs cross-scale representations through gated feature reconstruction and threshold-guided activation. Consequently, EFC aims to reduce the semantic gap between hierarchical features rather than merely reweighting existing feature responses. For UAV aerial object detection, EFC reduces the semantic gap between hierarchical features while preserving spatial information that is important for small-target localization. Thus, EFC provides more discriminative multi-scale representations for subsequent Transformer encoding and object prediction.

### 3.4. Feature Channel Attention Module

**Figure 4:**
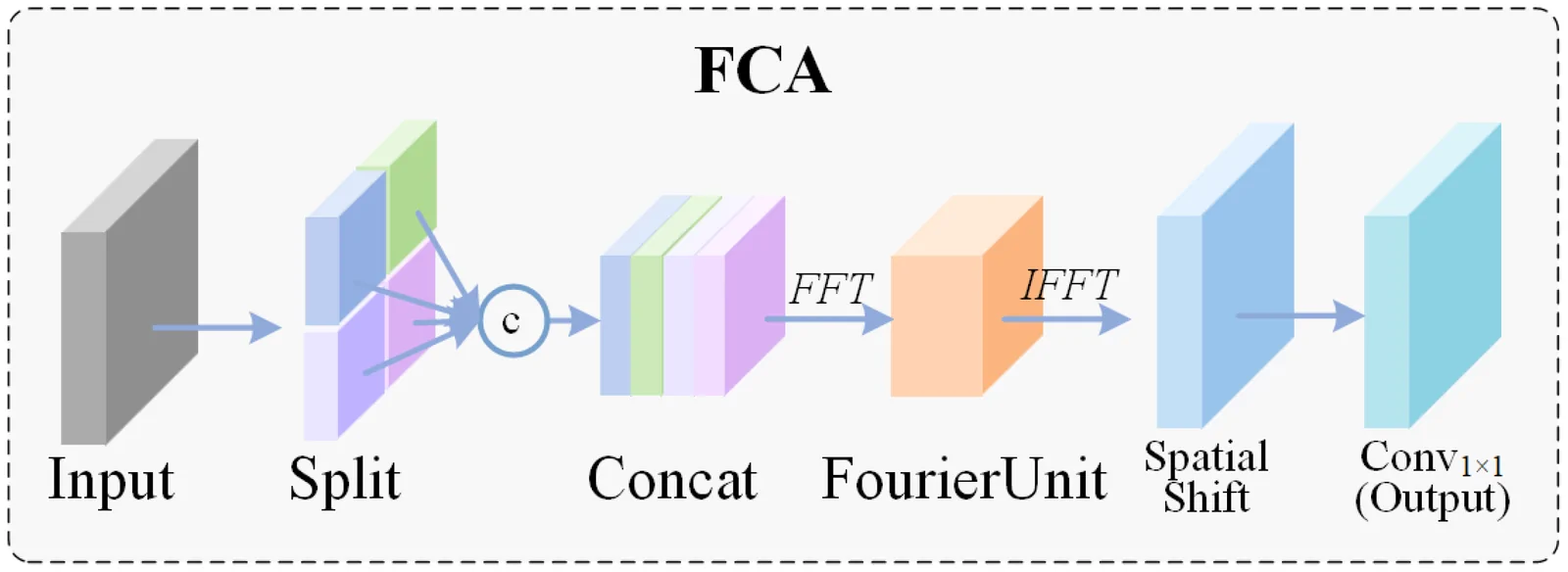
Schematic diagram of the feature channel attention module.

UAV aerial images often contain large and cluttered background regions, such as vegetation, shadows, and terrain textures. These background patterns can produce strong feature responses that interfere with target representation, particularly when small objects occupy only a limited portion of the image. Therefore, UAV object detection requires a feature enhancement mechanism that can selectively strengthen target-relevant responses while suppressing redundant background information. To address the background interference problem in UAV aerial object detection, we propose a Feature Channel Attention (FCA) module that performs adaptive channel-wise recalibration. Unlike conventional channel attention mechanisms that rely solely on global average pooling, FCA systematically integrates frequency-domain modeling and spatial-channel reorganization to capture both global contextual patterns and local discriminative cues. The overall architecture of the FCA module is illustrated in Figure 4.

Let the input feature map be denoted as $\;F\in R^{C\times H\times W}$, where  C, H, and W denote the number of channels, height, and width, respectively. The FCA module first applies a space-to-depth transformation, reorganizing spatial information into the channel dimension to preserve local context. The transformed feature map is represented as $\;\overset{ˆ}{F}\in R^{C^{\prime }\times H^{\prime }\times W^{\prime }}$:

(7) $${\overset{ˆ}{F}}_{c,h,w}=R\left(F\right),\;c=1,\ldots ,C^{\prime };h=1,\ldots ,H^{\prime };w=1,\ldots ,W^{\prime },$$

where R(⋅) denotes the deterministic space-to-depth reorganization operator.

The rearranged feature F is then mapped to the frequency domain via the two-dimensional Fourier transform, capturing global structural dependencies across channels:

(8) $$F=F_{2D}\overset{ˆ}{F},$$

(9) $$F^{\prime }c,h^{\prime },w^{\prime }=\sum {i=1}^{H^{\prime }}\sum_{j=1}^{W^{\prime }}W_{f}^{c,i,j}\cdot F_{c,i,j},$$

where $W_{f}\in R^{C^{\prime }\times H^{\prime }\times W^{\prime }}$ denotes a learnable complex-valued convolution kernel that modulates spectral components. The enhanced frequency-domain representation is then projected back to the spatial domain using the inverse Fourier transform:

(10) $$F_{f}=F_{2D}^{-1}F^{\prime },$$

To facilitate dynamic inter-channel interactions while maintaining spatial consistency, a spatial shift operation is applied to $F_{f}$. This operation redistributes local spatial information across channels:

(11) $$F_{s}=Shift\left(F_{f}\right),$$

where Shift(⋅) is a deterministic permutation operator that enhances channel-wise correlations and cross-channel contextual propagation.

Subsequently, a lightweight bottleneck convolution followed by a Sigmoid activation generates the channel attention map $A_{c}\in {\left[0,1\right]}^{C\times 1\times 1}$:

(12) $$A_{c}^{c}=\sigma \left(\sum_{c^{\prime }=1}^{C^{\prime }}W_{c}^{c,c^{\prime }}F_{s}^{c^{\prime },h,w}+b_{c}^{c}\right),\;c=1,\ldots ,C,$$

where $W_{c}$ and $b_{c\;}$ are learnable parameters, and  σ(⋅) denotes the sigmoid function. The attention map is then broadcast along spatial dimensions and applied to the original feature map to obtain the recalibrated output:

(13) $$F_{out}^{c,h,w}=F^{c,h,w}\cdot A_{c}^{c},$$

By integrating frequency-domain modeling with spatial-channel reorganization, FCA captures both global spectral characteristics and local discriminative cues. This design enables the network to emphasize task-relevant channels while suppressing redundant background responses, thereby improving the signal-to-noise ratio of target features. For UAV aerial object detection, this channel-wise refinement is particularly beneficial when small targets are surrounded by complex and visually similar backgrounds, as it helps preserve target-related information while reducing background interference.

### 3.5. Reinforced Attention Feedback Module

UAV aerial images often contain small, partially occluded, and densely distributed targets, whose localization depends strongly on fine-grained spatial cues such as local boundaries and textures. However, these cues can be weakened during successive Transformer encoding as features become increasingly semantic. Therefore, preserving informative intermediate spatial representations is important for maintaining accurate localization of small and occluded UAV targets.

In transformer-based object detection frameworks, multi-head self-attention mechanisms are central to refining the position-encoded feature representations, enabling the model to capture long-range dependencies and complex spatial relationships. Nevertheless, conventional attention modules often discard intermediate feature outputs after each layer, which can result in the loss of fine-grained spatial information that is crucial for accurately localizing small or partially occluded objects. To address this limitation, we propose the RAFM, which systematically reintroduces and enriches low-level attention outputs into subsequent layers. By recycling intermediate spatial representations during Transformer encoding, RAFM preserves fine-grained cues that are particularly important for localizing small and partially occluded UAV targets while maintaining computational efficiency. Formally, let $D_{out}^{\left(i-1\right)}\in R^{C\times H\times W}$ denote the output of the deformable attention (DA) in the (i−1)-th encoder layer. This feature map is first processed through a linear-convolution-linear (LCL) transformation, expressed as

(14) $${\overset{ˆ}{D}}^{\left(i-1\right)}=\sigma \left(W_{2}\;*\;Conv_{3\times 3}\left(W_{1}D_{out}^{\left(i-1\right)}+b_{1}\right)+b_{2}\right),$$

where $W_{1},W_{2}$ are learnable projection matrices for dimensional expansion and compression, $b_{1},b_{2}$ are biases, ∗ denotes convolution, and σ(⋅)  represents a non-linear activation function. The LCL operation first expands the channel dimension to increase representational capacity, then applies a 3 × 3 convolution to encode localized spatial correlations, and finally compresses the channels while introducing non-linear feature interactions. The enriched representation ${\overset{ˆ}{D}}^{\left(i-1\right)}$ is combined with the output of the feed-forward network (FFN) to form the input to the next DA block:

(15) $$D_{in}^{\left(i\right)}={\overset{ˆ}{D}}^{\left(i-1\right)}+FFN\left(D_{out}^{\left(i-1\right)}\right),$$

**Figure 5:**
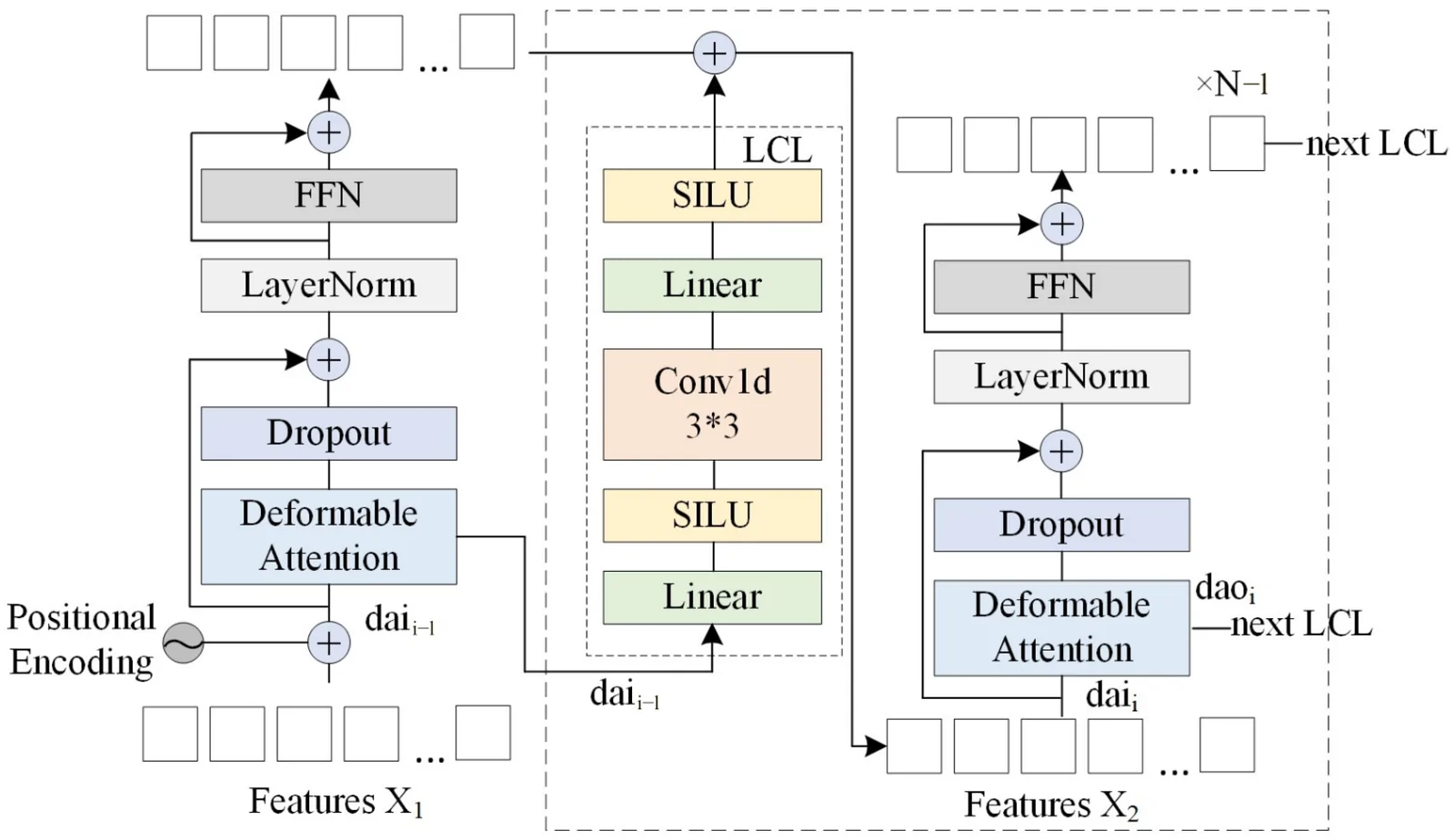
Structure of Reinforced Attention Feedback Module.

The overall structure of the RAFM is illustrated in Figure 5.

Within each deformable attention block, multi-scale feature maps are flattened and integrated with positional encodings to generate query embeddings $Q\in R^{N_{q}\times C}$. The deformable attention mechanism predicts a sparse set of offsets $\;\Delta p_{k}{k=f1}^{K}$ and attention weights $\alpha_{k}{k=1}^{K}$ for each query relative to its reference point $p_{0}$, where K is the number of sampled points. The output is then computed as

(16) $$D_{out}^{\left(i\right)}=\sum_{k=1}^{K}\alpha_{k}F\left(p_{0}+\Delta p_{k}\right),$$

where F(⋅) denotes the feature value at the sampled spatial location. By reintroducing the enriched low-level outputs ${\overset{ˆ}{D}}^{\left(i-1\right)}$, the RAFM enhances the representation of features across scales, ensuring that both small-scale textures and larger structural patterns are adequately preserved. To further improve cross-layer propagation and maintain feature coherence, RAFM optionally employs a multi-scale aggregation scheme, in which features from multiple preceding layers ${D_{out}^{\left(i-s\right)}}_{s=1}^{S}$ are aligned via learnable linear projections and summed:

(17) $$\tilde{D}{in}^{\left(i\right)}=\sum {s=1}^{S}W_{s}{\overset{ˆ}{D}}^{\left(i-s\right)}+FFN\left(D_{out}^{\left(i-1\right)}\right),$$

where S is the aggregation window size and $W_{s}$ are learnable weights that control the contribution of each previous layer. The aggregation window size S determines the receptive range for local feature aggregation. A small window may provide insufficient contextual information, whereas an excessively large window tends to smooth local structural details and introduces unnecessary computational overhead. Therefore, S is fixed to 3, which achieves a good trade-off between local spatial modeling capability and computational efficiency. This formulation allows the module to propagate both high-resolution spatial cues and deep semantic context, enhancing the network’s sensitivity to occluded or small objects without significantly increasing computational cost. In practice, the RAFM integrates seamlessly with the deformable attention mechanism, such that the enhanced input ${\tilde{D}}_{in}^{\left(i\right)}$ participates in offset prediction and attention weighting, effectively refining keypoint selection and attention distribution. Consequently, the module not only improves the fidelity of spatial representations across layers but also stabilizes training by mitigating the loss of informative intermediate features. By combining low-level feature recycling, multi-scale aggregation, and deformable attention, RAFM enables the network to capture fine-grained, high-resolution information alongside global context, substantially improving detection performance for small, occluded, or densely distributed objects in complex environments.

Relationship to Prior Work. Although RAFM shares the intuition of feature reuse with DenseNet, its objective differs fundamentally. DenseNet concatenates intermediate CNN features to improve feature propagation, whereas RAFM reinjects attention-enhanced representations into successive Transformer encoder layers after local spatial refinement. Therefore, RAFM strengthens deformable attention with enriched spatial cues instead of simply increasing feature reuse through dense connectivity.

### 3.6. Unified Query Supervision Loss

Existing DETR-based detectors usually improve query optimization from a single perspective. DN-DETR introduces denoising supervision to accelerate convergence by reconstructing perturbed ground-truth targets, whereas Group-DETR employs one-to-many assignment to increase positive supervision during training. Although these methods have demonstrated effectiveness, they optimize query learning independently and do not explicitly consider the complementary interactions among different supervision signals.

To address this limitation, the proposed UQSL establishes a unified query supervision paradigm that jointly integrates Hungarian matching, contrastive denoising supervision, and one-to-many assignment into a coordinated optimization framework. Rather than treating these objectives as isolated auxiliary tasks, UQSL enables them to collaboratively guide query evolution throughout decoder training, thereby improving supervision diversity, optimization stability, and representation quality.

Training transformer-based detection frameworks often suffer from slow convergence and instability, particularly when handling small or densely distributed objects. To address these challenges, we propose a UQSL that integrates multiple complementary supervision signals to guide query learning in a cohesive manner. Instead of relying solely on standard one-to-one matching, UQSL combines three distinct objectives—canonical bipartite matching, one-to-many supervision, and contrastive denoising—into a unified loss formulation, thereby improving convergence and enhancing robustness under occlusion and cluttered backgrounds.

(18) $$L1:1=\sum {l=1}^{L}H\left(P^{\left(l\right)},G_{V}\right)+\sum_{l=L+1}^{M}H\left(P^{\left(l\right)},G_{F}\right),$$

where H denotes the combined classification and localization loss (including GIoU), L is the index separating shallow and deep decoder layers, and M is the total number of decoder layers. This formulation ensures that early layers focus on partially visible objects while deeper layers supervise complete object reconstruction.

**Figure 6:**
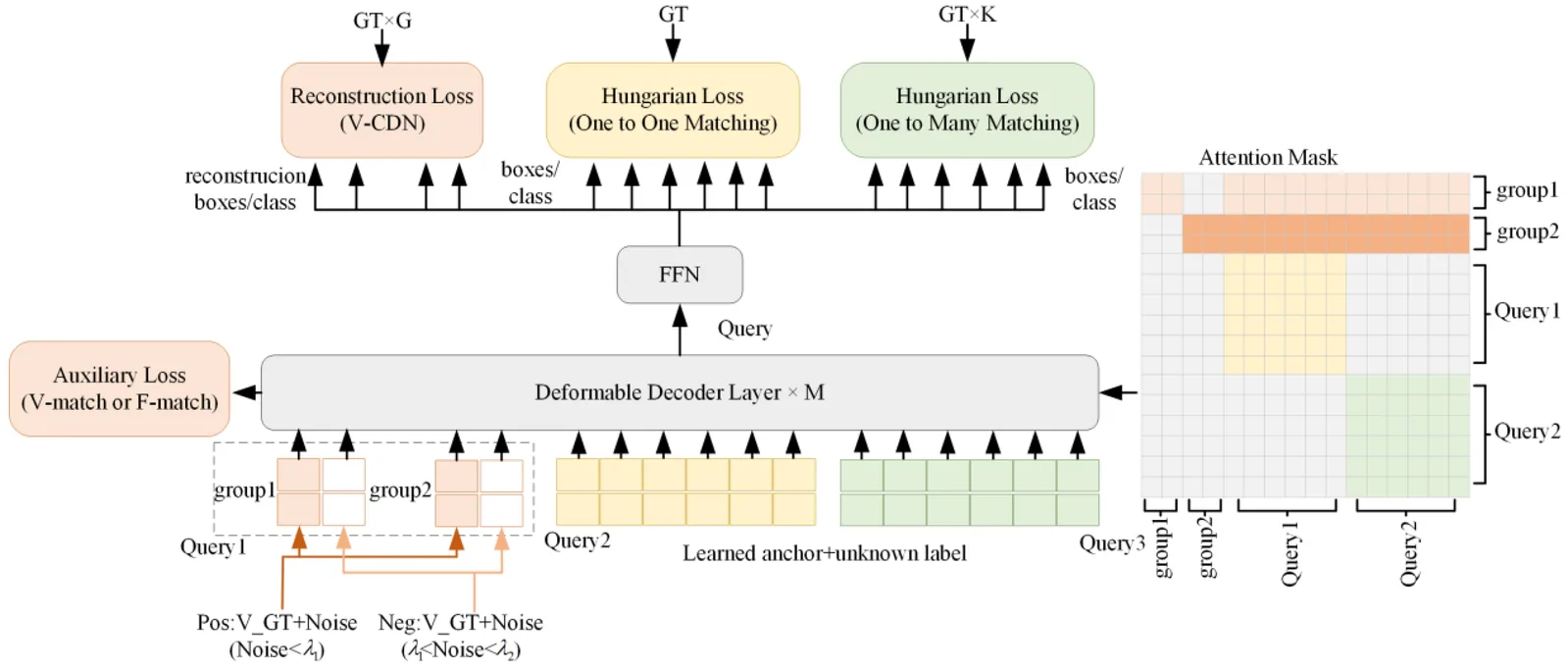
Structure of the hybrid decoding branches and hybrid decoding loss.

The overall structure of the hybrid decoding branches and hybrid decoding loss is illustrated in Figure 6.

To further enhance the model’s resilience to occlusion, we introduce a contrastive denoising reconstruction branch, in which queries are initialized with perturbed class labels and bounding boxes, forming positive-negative pairs. For a noisy query, the reconstruction objective is defined as:

(19) $$LCDN=\sum {l=1}^{M}\sum_{g=1}^{G}L\left(P_{g}^{\left(l\right)},G_{g}\right),$$

where G is the number of noisy groups, $P_{g}^{\left(l\right)}$ is the prediction corresponding to the g-th noisy query, and $G_{g}$ is its object. This encourages the network to recover full object representations from incomplete or corrupted inputs while suppressing false positives.

Finally, to mitigate the low positive-query ratio common in dense detection scenarios, a one-to-many matching branch is incorporated. Each ground-truth instance is replicated K times to increase positive query coverage, resulting in the loss:

(20) $$L1:K=\sum {l=1}^{L}H\left(P^{\left(l\right)},K\times G_{V}\right)+\sum_{l=L+1}^{M}H\left(P^{\left(l\right)},K\times G_{F}\right),$$

In all experiments, the one-to-many assignment number K is fixed to 5. This value provides sufficient positive supervision for query optimization while avoiding excessive duplicate assignments that may introduce optimization redundancy.

The final unified loss is then formulated as a summation of the three components:

(21) $$LUQSL=\lambda_{1}L1:1+\lambda_{2}LCDN+\lambda_{3}L1:K,$$

To quantitatively justify the use of equal weights for the three loss components, a sensitivity analysis was conducted on the validation set while keeping the network architecture, data partition, optimization strategy, and all other hyperparameters unchanged. Seven representative weighting configurations were evaluated by varying the relative contributions of the Hungarian matching, denoising, and one-to-many loss components. The corresponding results are presented in Table 1.

As shown in Table 1, the equal-weight configuration, W1, with $\lambda_{1}=\lambda_{2}=\lambda_{3}=1$, achieves the best overall performance, obtaining 86.34 ± 0.31% mAP50 and 64.46 ± 0.27% mAP50–95. All six non-equal configurations yield lower values for both evaluation metrics. The best-performing non-equal configuration is W4 (1,1,2), which achieves 86.20 ± 0.30% mAP50 and 64.29 ± 0.28% mAP50–95, corresponding to decreases of 0.14 and 0.17 percentage points, respectively, compared with W1. Moreover, assigning a substantially larger weight to an individual component can lead to further performance degradation. For example, W6 (1,3,1) achieves 85.95 ± 0.38% mAP50 and 63.94 ± 0.32% mAP50–95, which are 0.39 and 0.52 percentage points lower than the equal-weight configuration, respectively. These results provide direct numerical evidence that assigning a larger weight to any individual supervision component does not improve the overall detection performance. Instead, the equal-weight configuration provides the most favorable balance among the three complementary supervision objectives. Therefore, $\lambda_{1}=\lambda_{2}=\lambda_{3}=1$ is adopted as the default setting in all subsequent experiments. This setting allows Hungarian matching, contrastive denoising, and one-to-many supervision to contribute jointly to query optimization without introducing additional weighting factors or dataset-specific loss tuning.

It should be emphasized that the contribution of UQSL does not lie in the individual supervision components themselves, since Hungarian matching, denoising supervision, and one-to-many assignment have been explored in previous studies. Instead, the novelty originates from the unified optimization mechanism that coordinates these complementary supervision signals within a single query learning process. Specifically, Hungarian matching provides stable one-to-one supervision for accurate localization, contrastive denoising improves robustness against query perturbations, and one-to-many assignment enriches positive training signals for difficult samples. By jointly optimizing these objectives, UQSL promotes more diverse and discriminative query representations, leading to improved convergence behavior and stronger detection performance.

During training, structured masking is applied to prevent interference among branches: the contrastive denoising branch may attend to features from both matching branches, whereas one-to-one and one-to-many branches remain mutually isolated. This design allows each supervision signal to operate independently while benefiting from shared feature representations. By integrating multiple complementary objectives into a single unified framework, UQSL accelerates convergence, enhances gradient signals, and significantly improves localization accuracy, particularly in challenging object detection scenarios with occlusion, scale variation, and dense object distributions.

Relationship to Prior Work. DN-DETR accelerates convergence through denoising supervision, while Group-DETR increases positive samples via one-to-many assignment. In contrast, UQSL does not simply combine these objectives as independent auxiliary losses. Instead, it formulates them as a unified optimization framework in which complementary supervision signals jointly guide query evolution throughout decoder training. This coordinated optimization enables more consistent gradient propagation across decoder layers and improves optimization stability under dense object distributions. The detailed implementation procedure of the proposed MSF-DETR framework is presented in Algorithm 1.
**Algorithm 1.** Overall forward propagation and unified query supervision procedure of MSF-DETR  Input: UAV aerial image I; ground-truth annotations G  Output: Detection predictions Y; training loss $L_{UQSL}$  Hierarchical feature extraction: Extract multi-level backbone features             $\left\{S_{3},S_{4},S_{5}\right\}\leftarrow Backbone\left(I\right).$  Multi-scale feature projection: Generate the three feature levels used by the proposed framework:             $\left\{P_{3},P_{4},P_{5}\right\}\leftarrow Projection\left(\left\{S_{3},S_{4},S_{5}\right\}\right).$  Cross-scale feature selection: For each adjacent deep-shallow feature pair $\left(F_{d},F_{s}\right)$ derived from $\left\{P_{3},P_{4},P_{5}\right\}$, perform the following EFC operations.  Spatial alignment: Align the deep feature with the spatial resolution of the shallow feature:             $\begin{array}{cccc} & {\hat{F}}_{d}=\varphi \;\left(U\left(F_{d}\right)\right). & & \end{array}$  Joint feature embedding: Construct the cross-level joint representation:             $\begin{array}{cccc} & F_{joint}=\varphi \;\left(\left[F_{s},{\hat{F}}_{d}\right]\right). & & \end{array}$  Adaptive gating: Generate the spatially adaptive gating map:             $\begin{array}{cccc} & G=\sigma \;\left(\psi \left(F_{joint}\right)\right). & & \end{array}$  Dynamic feature reconstruction: Fuse the shallow and aligned deep features according to the learned gate:             $\begin{array}{cccc} & F_{rec}=G⊙F_{s}+\left(1-G\right)⊙{\hat{F}}_{d}. & & \end{array}$  Threshold-guided enhancement: Suppress weak responses using the learnable threshold τ:             $\begin{array}{cccc} & F_{enh}=ReLU\left(F_{rec}-\tau \right). & & \end{array}$  Residual refinement: Preserve fine-grained spatial information through the residual connection:             $\begin{array}{cccc} & F_{out}=F_{enh}+F_{s}. & & \end{array}$  Feature channel attention: Apply the FCA module to the enhanced multi-scale feature representation.  Space-to-depth reorganization:             $\begin{array}{cccc} & {\hat{F}}_{c,h,w}=R\left(F\right). & & \end{array}$  Frequency-domain transformation:             $\begin{array}{cccc} & F=F_{2D}\left(\hat{F}\right). & & \end{array}$  Frequency-domain feature modulation: Apply the learnable complex-valued spectral kernel $W_{f}$ according to Equation (10), obtaining the enhanced frequency representation $F^{\prime }$.  Inverse frequency transformation:             $\begin{array}{cccc} & F_{f}=F_{2D}^{-1}\left(F^{\prime }\right). & & \end{array}$  Spatial-channel interaction:             $\begin{array}{cccc} & F_{s}=Shift\left(F_{f}\right). & & \end{array}$  Channel attention generation: Generate the channel attention weights:             $\begin{array}{cccc} & A_{c}^{c}=\sigma \;\left(\sum_{c^{\prime }=1}^{C^{\prime }}W_{c}^{c,c^{\prime }}F_{s}^{c^{\prime },h,w}+b_{c}^{c}\right). & & \end{array}$  Channel recalibration:             $\begin{array}{cccc} & F_{out}^{c,h,w}=F^{c,h,w}\cdot A_{c}^{c}. & & \end{array}$  Transformer encoding: Feed the FCA-enhanced multi-scale features into the Transformer encoder and flatten the multi-scale representations to construct query embeddings Q.  RAFM feature feedback: For each Transformer encoder layer i, take the deformable-attention output $D_{out}^{\left(i-1\right)}$ and perform the LCL transformation:             $\begin{array}{cccc} & {\hat{D}}^{\left(i-1\right)}=\sigma \;\left(W_{2}\;*\;{Conv}_{3\times 3}\left(W_{1}D_{out}^{\left(i-1\right)}+b_{1}\right)+b_{2}\right). & & \end{array}$  Cross-layer feedback: Combine the enriched representation with the FFN output:             $\begin{array}{cccc} & D_{in}^{\left(i\right)}={\hat{D}}^{\left(i-1\right)}+\mathrm{FFN}\left(D_{out}^{\left(i-1\right)}\right). & & \end{array}$  Deformable attention: Generate query embeddings, reference points, sampling offsets, and attention weights, and compute:             $\begin{array}{cccc} & D_{out}^{\left(i\right)}=\sum_{k=1}^{K}\alpha_{k}F\left(p_{0}+\Delta p_{k}\right). & & \end{array}$  Multi-scale cross-layer aggregation: Aggregate the preceding enriched representations:             $\begin{array}{cccc} & {\tilde{D}}_{in}^{\left(i\right)}=\sum_{s=1}^{S}W_{s}{\hat{D}}^{\left(i-s\right)}+\mathrm{FFN}\left(D_{out}^{\left(i-1\right)}\right),S=3. & & \end{array}$  Query decoding: Feed the refined encoded features into the Transformer decoder to obtain the prediction sets             ${\left\{P^{\left(l\right)}\right\}}_{l=1}^{M}.$  One-to-one supervision: Perform Hungarian matching between the decoder predictions and the corresponding ground-truth targets and calculate:             $\begin{array}{cccc} & L_{1:1}=\sum_{l=1}^{L}H\left(P^{\left(l\right)},G_{V}\right)+\sum_{l=L+1}^{M}H\left(P^{\left(l\right)},G_{F}\right). & & \end{array}$  Contrastive denoising supervision: Construct perturbed/noisy query targets from the ground-truth annotations, generate noisy query groups, and compute:             $\begin{array}{cccc} & L_{CDN}=\sum_{l=1}^{M}\sum_{g=1}^{G}L\left(P_{g}^{\left(l\right)},G_{g}\right). & & \end{array}$  One-to-many supervision: Replicate each ground-truth instance K times (K=5) and perform one-to-many assignment:             $\begin{array}{cccc} & L_{1:K}=\sum_{l=1}^{L}H\left(P^{\left(l\right)},K\times G_{V}\right)+\sum_{l=L+1}^{M}H\left(P^{\left(l\right)},K\times G_{F}\right). & & \end{array}$  Unified query supervision loss: Combine the three supervision objectives:             $\begin{array}{cccc} & L_{UQSL}=L_{1:1}+L_{CDN}+L_{1:K}. & & \end{array}$  Parameter optimization: During training, back-propagate $L_{UQSL}$ to jointly optimize the trainable parameters of EFC, FCA, RAFM, the Transformer decoder, and the query-supervision branches.  Inference: During inference, omit the supervision branches and directly obtain the final object detection results from the Transformer decoder:             $Y\leftarrow \mathrm{Decoder}\left({\tilde{D}}_{in}\right).$  Return: Return the final detection predictions Y and, during training, the unified loss $L_{UQSL}$.

### 3.7. Comparison with Closest Existing Methods

To more clearly distinguish the methodological contributions of MSF-DETR from closely related approaches, a component-level comparison is presented in Table 2. Rather than comparing only the overall detector architectures, the comparison focuses on the specific operations and optimization mechanisms associated with multi-scale feature fusion, frequency-aware channel modeling, attention feedback, and query supervision. This analysis also clarifies which basic concepts have been established in previous studies and which specific formulations are introduced in the present work.

The comparison shows that the individual concepts incorporated into MSF-DETR are related to established research directions, but their specific operations and coordination differ from those of the closest existing approaches. For cross-scale representation, BiFPN performs repeated bidirectional feature aggregation using learnable fusion weights. EFC, in contrast, establishes spatially aligned cross-level representations and generates spatially adaptive gating responses before performing gated feature reconstruction and threshold-guided activation. Therefore, its contribution is not multi-scale fusion itself, but the adaptive reconstruction of heterogeneous shallow and deep representations for UAV aerial targets with different scales.

For frequency-aware feature modeling, FcaNet has demonstrated the effectiveness of frequency-domain information for channel attention. MSF-DETR does not claim frequency-domain modeling itself as a novel concept. Instead, FCA combines frequency-domain modulation with space-to-depth reorganization, spatial shifting, and lightweight channel recalibration, thereby jointly exploiting global frequency characteristics and local spatial-channel information. This formulation is intended to improve target-background discrimination in cluttered UAV aerial scenes.

For Transformer feature propagation, existing feature reuse strategies mainly focus on reusing intermediate representations through dense connectivity or skip connections. RAFM differs by operating directly on intermediate deformable-attention outputs within successive Transformer encoder layers. The attention responses are enriched through the LCL transformation and fed back to subsequent attention blocks, allowing informative spatial responses from earlier encoding stages to participate in later attention computation. Thus, the distinctive operation of RAFM is attention-level feedback rather than feature reuse in general.

For query optimization, DN-DETR introduces denoising queries to improve the stability of Hungarian matching, while Group-DETR introduces group-wise one-to-many assignment to provide additional positive supervision. Co-DETR further employs auxiliary heads and one-to-many assignments to generate positive queries for collaborative training. UQSL builds upon these established supervision concepts but does not claim denoising or one-to-many assignment as independent innovations. Instead, it coordinates one-to-one Hungarian matching, denoising supervision, and one-to-many assignment within a unified query-level optimization objective. This formulation is designed to provide richer and more stable supervision for densely distributed UAV targets while retaining the final set-prediction paradigm.

Overall, the three proposed architectural modules and the query optimization mechanism address complementary aspects of UAV aerial object detection. EFC targets scale-dependent feature inconsistency, FCA improves discrimination under cluttered backgrounds, RAFM preserves informative spatial details during Transformer encoding, and UQSL stabilizes query learning in dense scenes. Therefore, the contribution of MSF-DETR lies not in introducing isolated versions of concepts that have already appeared in the literature, but in developing specific operations and a coordinated optimization strategy around the characteristic scale variation, background interference, spatial-detail degradation, and dense target distribution of UAV aerial imagery.

## 4. Experiments

To evaluate the effectiveness and robustness of the proposed MSF-DETR, extensive experiments are conducted on two representative optical remote sensing and aerial object detection datasets, DIOR and DOTA. Standard evaluation metrics, including mAP50 and mAP50–95, are adopted to comprehensively assess detection performance. Comparative experiments with multiple representative detection methods are carried out to analyze the advantages of the proposed framework. In addition, ablation studies are performed to investigate the contribution of each component. This section first introduces the experimental settings and datasets, followed by detailed analyses of ablation results and comparisons with existing methods.

### 4.1. Experimental Settings

The experimental environment is based on PyTorch (v2.3.0) and Python (v3.8.10). The hardware includes an NVIDIA RTX 4060 Ti GPU (NVIDIA Corporation, Santa Clara, CA, USA), an Intel Core i5-12400F CPU (Intel Corporation, Santa Clara, CA, USA), and CUDA (v12.1). The batch size is set to 10, with 100 training epochs and a learning rate of 1 × 10^−4^, with a cosine annealing schedule that gradually reduces the rate to 1 × 10^−6^ toward the end of training. The AdamW optimizer is adopted with a weight decay of 1 × 10^−4^ and momentum parameters β1 = 0.9, β2 = 0.999. The warm-up phase lasts for the first 5 epochs, during which the learning rate linearly increases from zero to its maximum value to stabilize the optimization process.

Before training, all input images are resized to 640 × 640 pixels. The corresponding annotations are transformed consistently with the image resizing operation to preserve the spatial correspondence between input images and object labels. For the DOTA dataset, the original high-resolution images are cropped into fixed-size patches during preprocessing, while the official training, validation, and testing partitions remain unchanged. The corresponding object annotations are converted from the original quadrilateral representation into the annotation format required by the detector. During training, the data augmentation strategy is kept consistent across all compared methods to improve generalization and ensure a fair comparison. No stochastic data augmentation is applied during validation or testing.

The proposed framework is initialized based on the RT-DETR architecture, and the newly introduced modules are initialized using the corresponding default parameter initialization strategy. The Transformer decoder retains the fixed set of learnable object queries used by the RT-DETR framework. Gradient clipping is not applied during training. Mixed-precision training is enabled using FP16 automatic mixed precision. To improve reproducibility, each ablation configuration is independently trained and evaluated ten times using different random seeds, and the reported results correspond to the average performance over these ten runs. This protocol reduces the influence of stochastic optimization and random initialization on the reported results.

The key hyperparameters of the proposed framework are determined through controlled preliminary experiments on the validation set. Specifically, the threshold τ\tau, the aggregation window size SS in RAFM, and the one-to-many matching number KK in UQSL are varied individually while the network architecture, dataset partition, optimization strategy, and other hyperparameters remain unchanged. Several candidate settings are evaluated to examine their effects on overall detection performance. Based on these preliminary experiments, τ = 0.1, S = 3, and K = 5 are selected because they provide the most favorable overall detection performance. In particular, S = 3 provides a suitable balance between contextual aggregation and computational cost; smaller values provide insufficient contextual information, whereas larger values introduce additional redundancy and computational overhead. This selection is consistent with the design motivation of RAFM described in Section 3.5. For UQSL, K = 5 provides sufficient one-to-many positive supervision without introducing excessive redundant assignments. The three loss components in UQSL are assigned equal weights because preliminary experiments show that their contributions remain relatively balanced during optimization, and additional manually tuned weighting coefficients do not provide consistent performance improvements. Once selected, these hyperparameters are fixed throughout the subsequent experiments and are not individually adjusted for DIOR or DOTA, thereby avoiding dataset-specific parameter tuning and providing a consistent basis for evaluating the general applicability of the proposed framework.

During inference, the same image resizing procedure is applied as during training, and the input resolution is fixed at 640 × 640 pixels. No stochastic data augmentation is introduced during testing. Since MSF-DETR follows the end-to-end RT-DETR detection paradigm, the final predictions are directly obtained from the decoder without conventional non-maximum suppression. For DOTA, predictions generated from the cropped image patches are mapped back to the original image coordinates and merged before final evaluation. The official DOTA dataset partition is retained throughout the experiments, while image cropping is treated solely as a preprocessing operation.

For inference-speed evaluation, MSF-DETR is tested with a batch size of 8 at an input resolution of 640 × 640 pixels on the NVIDIA RTX 4060 Ti GPU. Inference is performed using FP16 automatic mixed precision with gradient computation disabled. The trained model checkpoint is loaded before timing. Subsequently, 100 consecutive inference iterations are measured using CUDA-synchronized timing. The same measurement protocol is used when comparing the inference speed of MSF-DETR with other reproduced models.

The deformable attention mechanism does not rely on manually specified spatial control points. Instead, the reference points, sampling offsets, and attention weights are generated adaptively by the deformable-attention mechanism from the learned feature representations. This allows the spatial sampling locations to be learned jointly with the detection objective rather than manually predefined. In MSF-DETR, RAFM further refines the feature representations passed to the subsequent deformable-attention layers.

All experiments reported in this paper are conducted under the same training configuration to ensure fair and consistent comparisons among different methods. Unless otherwise stated, all compared methods use the same dataset partitions, input resolution, training epochs, batch size, optimizer, and learning-rate schedule. Each configuration is trained and evaluated under the same experimental protocol, while repeated training with different random seeds is used for the ablation experiments. This unified protocol reduces the influence of differences in experimental conditions and provides a more reliable assessment of the contribution of the proposed components.

### 4.2. Datasets

To comprehensively evaluate the effectiveness and generalization capability of the proposed MSF-DETR, experiments are conducted on two widely used benchmarks for optical remote sensing and aerial object detection, namely DIOR and DOTA. These two datasets differ substantially in image source, object scale, annotation format, scene complexity, and category distribution, thereby providing complementary evaluation scenarios for assessing the robustness of aerial object detectors. In this section, the descriptions of the two datasets strictly follow their original publications and official protocols. Any image cropping, patch generation, or annotation-format conversion used during implementation is regarded as a preprocessing operation and is not treated as an alternative official dataset split.

DIOR is a large-scale benchmark for object detection in optical remote sensing images, proposed by Li et al. It contains 23,463 images and 192,472 annotated object instances covering 20 object categories, including airplane, airport, baseball field, basketball court, bridge, chimney, dam, expressway service area, expressway toll station, golf course, ground track field, harbor, overpass, ship, stadium, storage tank, tennis court, train station, vehicle, and windmill. The images in DIOR are collected under diverse imaging conditions, spatial resolutions, seasons, weather conditions, and scene backgrounds, resulting in pronounced intra-class variations and inter-class similarities. Therefore, DIOR provides a challenging benchmark for evaluating the ability of detection models to handle scale variation, complex backgrounds, and category-level ambiguity in optical remote sensing imagery. In this work, the predefined dataset partition is adopted for training, validation, and testing, ensuring that all experimental results are obtained under a consistent and reproducible evaluation protocol.

DOTA-v1.0 is a large-scale dataset for object detection in aerial images, introduced by Xia et al. Unlike traffic-specific aerial datasets, DOTA is designed for general-purpose aerial object detection and contains images collected from different sensors and platforms. It consists of 2806 large-scale aerial images and 188,282 annotated object instances across 15 common categories, including plane, baseball diamond, bridge, ground track field, small vehicle, large vehicle, ship, tennis court, basketball court, storage tank, soccer-ball field, roundabout, harbor, swimming pool, and helicopter. Each object instance is annotated with an oriented quadrilateral bounding box, which enables more accurate localization of objects with arbitrary orientations. Due to its large image size, dense object distribution, significant scale variation, and arbitrary object orientation, DOTA is particularly suitable for evaluating detection robustness in challenging aerial scenes.

Following the official DOTA-v1.0 protocol, the dataset is officially divided into training, validation, and testing subsets, accounting for one-half, one-sixth, and one-third of the entire dataset, respectively. In this study, the official dataset partition is adopted without modification to ensure consistency with the standard evaluation protocol and to facilitate fair comparisons with existing methods. Since the original DOTA images have very high spatial resolutions, they are cropped into fixed-size patches only during the data preprocessing stage for model training and inference, while the official dataset partition remains unchanged throughout the experiments.

### 4.3. Comparison with Different Methods

To evaluate the effectiveness and robustness of the proposed MSF-DETR, we compared it with a series of representative state-of-the-art detection methods on the DIOR and DOTA datasets, as shown in Table 3 and Table 4, respectively. To reduce the influence of random factors during model training, each experiment was independently repeated ten times under the same experimental settings. The reported results were obtained by averaging the performance over the ten repeated experiments. In addition to detection accuracy, the computational complexity and inference efficiency of the compared methods were evaluated in terms of the number of parameters (Params), floating-point operations (GFLOPs), and inference speed (FPS) under the same input resolution and hardware environment.

Table 3 and Table 4 present the quantitative comparison results of MSF-DETR with a variety of CNN-based, YOLO-based, and Transformer-based detectors on the DIOR and DOTA datasets, respectively. In addition to conventional detectors, several representative Transformer-based methods, including DAB-DETR, Group-DETR, Co-DETR, and RT-DETR, are included to provide a comprehensive evaluation of the proposed framework.

On the DIOR dataset, MSF-DETR achieves the highest detection accuracy among the compared methods, reaching 86.3% mAP50 and 64.4% mAP50–95. Compared with the RT-DETR baseline, the proposed method improves mAP50 from 83.5% to 86.3% and mAP50–95 from 61.8% to 64.4%. Compared with recent Transformer-based detectors, including DAB-DETR, Group-DETR, and Co-DETR, MSF-DETR achieves higher detection accuracy across both evaluation metrics. Specifically, the proposed framework improves mAP50 by 3.6% over DAB-DETR and by 2.2% and 1.1% over Group-DETR and Co-DETR, respectively. These results suggest that the proposed EFC, FCA, RAFM, and UQSL modules improve multi-scale feature interaction and spatial representation, leading to more discriminative feature learning for UAV aerial imagery.

Compared with representative YOLO-based detectors, MSF-DETR also achieves higher detection accuracy. Although advanced variants such as YOLO-GE, RFAG-YOLO, and YOLO-MARS achieve competitive performance, their detection accuracy remains lower than that of the proposed method. Overall, the comparison results indicate that the proposed Transformer-based framework provides improved modeling of long-range dependencies and multi-scale contextual information.

On the DOTA dataset, which presents greater challenges due to dense object distributions, large-scale variations, and complex backgrounds, MSF-DETR also achieves the highest detection accuracy among the compared methods. Specifically, the proposed method obtains 77.2% mAP50 and 48.8% mAP50–95, improving upon the RT-DETR baseline by 4.7% and 4.6%, respectively. Compared with DAB-DETR, Group-DETR, and Co-DETR, MSF-DETR improves mAP50 by 2.6%, 1.9%, and 1.1%, respectively. Similar improvements are observed for mAP50–95. These results suggest that the proposed framework maintains good generalization capability under challenging aerial imaging scenarios characterized by dense object distributions, substantial scale variations, and complex backgrounds.

Compared with the results on DIOR, larger performance gains are observed on the DOTA dataset, indicating that the proposed modules are well suited to challenging UAV aerial imaging scenarios. The adaptive cross-scale feature interaction introduced by EFC improves semantic consistency across different feature levels, while RAFM enhances the preservation of fine-grained spatial information during Transformer encoding. Together with FCA and UQSL, these components contribute to the observed improvements in feature representation and query optimization, resulting in consistent detection performance across different datasets.

From the perspective of computational efficiency, the proposed MSF-DETR introduces a moderate computational overhead while achieving substantial improvements in detection accuracy. On the DIOR dataset, MSF-DETR contains 44.6M parameters and requires 91.4 GFLOPs, compared with 42.5M parameters and 85.1 GFLOPs for RT-DETR. Thus, the proposed method introduces only 2.1M additional parameters and 6.3 GFLOPs. Meanwhile, the inference speed decreases from 81.3 FPS to 73 FPS. Despite this moderate reduction in inference speed, MSF-DETR improves mAP50 from 83.5% to 86.3% and mAP50–95 from 61.8% to 64.4%.

A similar trend is observed on the DOTA dataset. MSF-DETR maintains 44.6M parameters, 91.4 GFLOPs, and an inference speed of 73 FPS, while improving mAP50 from 72.5% to 77.2% and mAP50–95 from 44.2% to 48.8% compared with RT-DETR. These results demonstrate that the additional computational cost introduced by the proposed EFC, FCA, RAFM, and UQSL modules contributes to consistent improvements in detection performance rather than merely increasing model complexity.

Although lightweight YOLO-based detectors generally achieve higher inference speeds and lower computational costs, their detection accuracy is also lower than that of MSF-DETR. In contrast, MSF-DETR maintains a real-time inference speed of 73 FPS while achieving the highest detection accuracy among the compared methods. Therefore, the proposed framework provides a favorable trade-off between detection accuracy and computational efficiency, demonstrating that the performance gains obtained from the additional computational cost are practically meaningful for aerial object detection applications.

### 4.4. Ablation Experiment

To comprehensively evaluate the effectiveness and complementarity of the proposed components, ablation experiments are conducted based on the RT-DETR baseline on the DIOR and DOTA datasets. Specifically, EFC, FCA, RAFM, and UQSL are progressively introduced into the baseline framework, and both individual and combined configurations are evaluated. In addition to the overall detection performance, APs, APm, and APl are reported to investigate the effectiveness of the proposed method for objects with different scales. To further assess the robustness and reproducibility of the experimental results, each ablation configuration is independently trained 10 times using different random initializations, and the results are reported as mean ± standard deviation. This statistical protocol reduces the influence of random initialization and provides a more reliable evaluation of the proposed components. The experimental results are presented in Table 5 and Table 6.

As shown in Table 5, the RT-DETR baseline on the DIOR dataset achieves 83.42 ± 0.37% mAP50 and 61.76 ± 0.29% mAP50–95, with APs, APm, and APl of 31.27 ± 0.48%, 60.71 ± 0.42%, and 72.84 ± 0.46%, respectively. The baseline model contains 42.5M parameters and requires 85.1 GFLOPs. The relatively low APs compared with APm and APl indicates that detecting small objects remains a challenging problem in aerial imagery.

When EFC is introduced individually, the mean mAP50 and mAP50–95 increase to 84.16 ± 0.32% and 63.06 ± 0.36%, respectively, corresponding to improvements of 0.74 and 1.30 percentage points over the baseline. Meanwhile, APs, APm, and APl increase to 32.73 ± 0.45%, 61.94 ± 0.47%, and 73.92 ± 0.51%, yielding gains of 1.46, 1.23, and 1.08 percentage points, respectively. These consistent improvements across different object scales indicate that EFC effectively enhances cross-scale feature interaction and strengthens the representation of objects with different spatial extents. The computational cost increases moderately from 85.1 to 86.6 GFLOPs, while the parameter count increases from 42.5M to 43.0M.

The individual introduction of FCA further improves the detection performance, achieving 84.63 ± 0.41% mAP50 and 62.58 ± 0.31% mAP50–95. The corresponding APs, APm, and APl reach 33.64 ± 0.51%, 61.43 ± 0.39%, and 73.62 ± 0.43%, respectively. Compared with the baseline, FCA improves APs by 2.37 percentage points and mAP50 by 1.21 percentage points. The relatively larger improvement in APs demonstrates that channel-wise feature recalibration can enhance the discriminative capability of object-related features, which is particularly beneficial for small objects with limited visual information. The introduction of FCA increases the computational cost to 90.7 GFLOPs, with 42.9M parameters.

RAFM achieves 84.86 ± 0.34% mAP50 and 63.27 ± 0.32% mAP50–95 when introduced individually. APs, APm, and APl reach 34.14 ± 0.43%, 62.16 ± 0.44%, and 74.15 ± 0.48%, respectively. Compared with the baseline, RAFM improves APs, APm, and APl by 2.87, 1.45, and 1.31 percentage points, respectively. Among the individual feature enhancement modules, RAFM produces the largest improvement in APs, indicating that it is particularly effective in preserving fine-grained spatial information during feature encoding. This property is important for aerial object detection because small targets often contain only limited spatial and semantic cues. The corresponding computational cost is 88.4 GFLOPs and the parameter count is 44.1M.

When UQSL is introduced independently, the model achieves 84.08 ± 0.38% mAP50 and 63.58 ± 0.35% mAP50–95. APs, APm, and APl reach 32.57 ± 0.49%, 62.46 ± 0.41%, and 74.91 ± 0.45%, respectively. Compared with the baseline, UQSL improves APs by 1.30 percentage points, APm by 1.75 percentage points, and APl by 2.07 percentage points. Notably, UQSL provides the largest individual improvement in APl, suggesting that improved query-level supervision can provide more effective optimization signals for object queries and facilitate the learning of high-quality object representations. Since UQSL does not introduce additional feature extraction operations, the computational complexity and parameter count remain unchanged at 85.1 GFLOPs and 42.5M, respectively.

When EFC and FCA are jointly integrated, the model achieves 85.35 ± 0.35% mAP50 and 63.38 ± 0.30% mAP50–95, with APs, APm, and APl reaching 34.93 ± 0.46%, 62.76 ± 0.43%, and 74.92 ± 0.47%, respectively. Compared with the baseline, the combined configuration improves APs, APm, and APl by 3.66, 2.05, and 2.08 percentage points, respectively. The simultaneous improvements across all object scales demonstrate that cross-scale feature interaction and channel-wise feature recalibration provide complementary feature enhancement capabilities. The computational cost increases to 91.6 GFLOPs, while the parameter count increases to 43.3M.

Further incorporating RAFM into the EFC + FCA configuration leads to additional improvements. The resulting model achieves 85.63 ± 0.33% mAP50 and 64.07 ± 0.28% mAP50–95, while APs, APm, and APl increase to 35.94 ± 0.50%, 63.43 ± 0.45%, and 75.46 ± 0.49%, respectively. Relative to the baseline, the gains reach 4.67, 2.72, and 2.62 percentage points for APs, APm, and APl, respectively. In particular, APs increases from 31.27% to 35.94%, demonstrating that the combination of cross-scale feature interaction, channel recalibration, and fine-grained spatial enhancement provides progressively stronger representations for small objects. This configuration requires 88.9 GFLOPs and 44.4M parameters.

Finally, after incorporating UQSL, the complete framework achieves the best mean performance across all reported accuracy metrics. Specifically, APs, APm, and APl reach 38.06 ± 0.47%, 64.17 ± 0.40%, and 76.94 ± 0.52%, respectively, while mAP50 and mAP50–95 reach 86.34 ± 0.31% and 64.46 ± 0.27%. Compared with the RT-DETR baseline, the complete framework improves APs by 6.79 percentage points, APm by 3.46 percentage points, APl by 4.10 percentage points, mAP50 by 2.92 percentage points, and mAP50–95 by 2.70 percentage points. The particularly notable improvement in APs confirms that the proposed framework is highly effective in enhancing the detection of small objects in aerial imagery.

The standard deviations reported in Table 5 are relatively small across all configurations, indicating that the observed performance improvements are not strongly dependent on a particular random initialization. For the complete model, the standard deviations of APs, APm, APl, mAP50, and mAP50–95 are 0.47, 0.40, 0.52, 0.31, and 0.27 percentage points, respectively. These relatively low variations across 10 independent training runs provide additional evidence for the stability and reproducibility of the proposed framework.

In terms of computational complexity, the complete model requires 91.4 GFLOPs and 44.6M parameters, compared with 85.1 GFLOPs and 42.5M parameters for the RT-DETR baseline. Thus, the proposed framework introduces an additional 6.3 GFLOPs and 2.1M parameters, corresponding to increases of approximately 7.4% and 4.9%, respectively. Considering the improvements across all object scales and the relatively small variation across independent runs, the proposed framework achieves a favorable trade-off between detection accuracy, robustness, and computational cost.

To further evaluate the generalization capability of the proposed components, the same ablation protocol is applied to the DOTA dataset, with the results presented in Table 6. The RT-DETR baseline achieves 72.43 ± 0.47% mAP50 and 44.14 ± 0.39% mAP50–95, with APs, APm, and APl of 20.43 ± 0.59%, 43.31 ± 0.56%, and 59.18 ± 0.53%, respectively. The baseline contains 42.5M parameters and requires 85.1 GFLOPs. The lower performance across all object scales compared with DIOR indicates that DOTA presents a more challenging aerial detection environment.

After introducing EFC, mAP50 and mAP50–95 increase to 73.76 ± 0.43% and 45.24 ± 0.41%, respectively. APs, APm, and APl improve to 21.94 ± 0.55%, 44.52 ± 0.51%, and 60.31 ± 0.57%, corresponding to gains of 1.51, 1.21, and 1.13 percentage points over the baseline. These consistent improvements across different object scales demonstrate that EFC can enhance cross-scale feature interaction under the different image distributions and complex object arrangements of DOTA. The computational cost increases from 85.1 to 86.6 GFLOPs, while the parameter count increases from 42.5M to 43.0M.

The FCA module independently achieves 73.18 ± 0.46% mAP50 and 44.87 ± 0.38% mAP50–95. Its APs, APm, and APl reach 21.46 ± 0.52%, 43.91 ± 0.57%, and 59.84 ± 0.50%, respectively. Compared with the baseline, FCA improves APs by 1.03 percentage points and mAP50 by 0.75 percentage points. Although its overall improvement is relatively moderate, the gain in APs indicates that FCA contributes to extracting more discriminative channel information, which is beneficial for small targets in complex aerial scenes. The corresponding computational cost is 90.7 GFLOPs with 42.9M parameters.

When RAFM is introduced individually, mAP50 and mAP50–95 increase to 73.59 ± 0.42% and 45.06 ± 0.37%, respectively. APs, APm, and APl reach 22.26 ± 0.58%, 44.42 ± 0.53%, and 60.21 ± 0.55%, respectively. The improvement in APs reaches 1.83 percentage points, which is the largest gain among the individual feature enhancement modules. This result is consistent with the observations on DIOR and further demonstrates that RAFM is effective in preserving fine-grained spatial information during Transformer encoding. The corresponding computational cost is 88.4 GFLOPs and the parameter count is 44.1M.

The individual introduction of UQSL increases mAP50 to 74.03 ± 0.45% and mAP50–95 to 45.65 ± 0.40%. APs, APm, and APl reach 21.73 ± 0.54%, 44.87 ± 0.50%, and 60.94 ± 0.52%, respectively. In particular, APl increases by 1.76 percentage points over the baseline, representing the largest individual improvement for large objects. This result suggests that improved query-level supervision can provide more consistent optimization signals and improve object-query learning. Similar to the DIOR experiments, UQSL does not introduce additional computational overhead, and the complexity remains at 85.1 GFLOPs with 42.5M parameters.

When EFC and FCA are jointly integrated, the model achieves 74.68 ± 0.39% mAP50 and 46.27 ± 0.34% mAP50–95. APs, APm, and APl increase to 23.14 ± 0.56%, 45.61 ± 0.54%, and 61.43 ± 0.58%, respectively. Compared with the baseline, the gains reach 2.71, 2.30, and 2.25 percentage points for APs, APm, and APl, respectively. The improvements across all object scales demonstrate that EFC and FCA provide complementary feature enhancement effects. This configuration requires 91.6 GFLOPs and 43.3M parameters.

When RAFM is further introduced, the model achieves 75.08 ± 0.41% mAP50 and 46.57 ± 0.36% mAP50–95, while APs, APm, and APl increase to 24.03 ± 0.61%, 46.02 ± 0.52%, and 61.91 ± 0.56%, respectively. Compared with the baseline, the improvements are 3.60 percentage points for APs, 2.71 percentage points for APm, and 2.73 percentage points for APl. These results demonstrate that the three feature enhancement modules jointly improve the representation capability of the detector across different object scales. The corresponding computational cost is 88.9 GFLOPs and the parameter count is 44.4M.

After incorporating UQSL into the complete framework, the model achieves the best mean performance on all reported accuracy metrics. APs, APm, and APl increase to 26.14 ± 0.57%, 48.32 ± 0.60%, and 64.71 ± 0.63%, respectively, while mAP50 and mAP50–95 reach 77.16 ± 0.38% and 48.74 ± 0.35%. Compared with the RT-DETR baseline, the complete framework improves APs by 5.71 percentage points, APm by 5.01 percentage points, APl by 5.53 percentage points, mAP50 by 4.73 percentage points, and mAP50–95 by 4.60 percentage points. The consistent gains across small-, medium-, and large-scale objects indicate that the proposed framework improves the overall representation capability of the detector rather than optimizing performance for a specific object scale.

The improvement in small-object detection is particularly notable. APs increases from 20.43 ± 0.59% for the baseline to 26.14 ± 0.57% for the complete framework, corresponding to a gain of 5.71 percentage points. This result demonstrates that the combination of feature enhancement and query-level supervision can effectively recover and exploit fine-grained information that is difficult to capture in challenging aerial scenes. At the same time, the improvements of 5.01 and 5.53 percentage points in APm and APl indicate that the proposed method also maintains strong representation capability for medium- and large-scale objects.

The standard deviations on DOTA are also relatively small, particularly for the overall detection metrics. For the complete framework, the standard deviations of APs, APm, APl, mAP50, and mAP50–95 are 0.57, 0.60, 0.63, 0.38, and 0.35 percentage points, respectively. These results indicate that the complete framework maintains stable performance across 10 independent training runs despite the greater difficulty of the DOTA dataset.

Regarding computational complexity, the complete model increases the computational cost from 85.1 to 91.4 GFLOPs and the parameter count from 42.5M to 44.6M. This corresponds to increases of approximately 7.4% in GFLOPs and 4.9% in parameters. Importantly, the complete framework achieves substantial improvements in both mAP50 and mAP50–95 on DOTA while introducing only moderate additional computational overhead.

**Figure 7:**
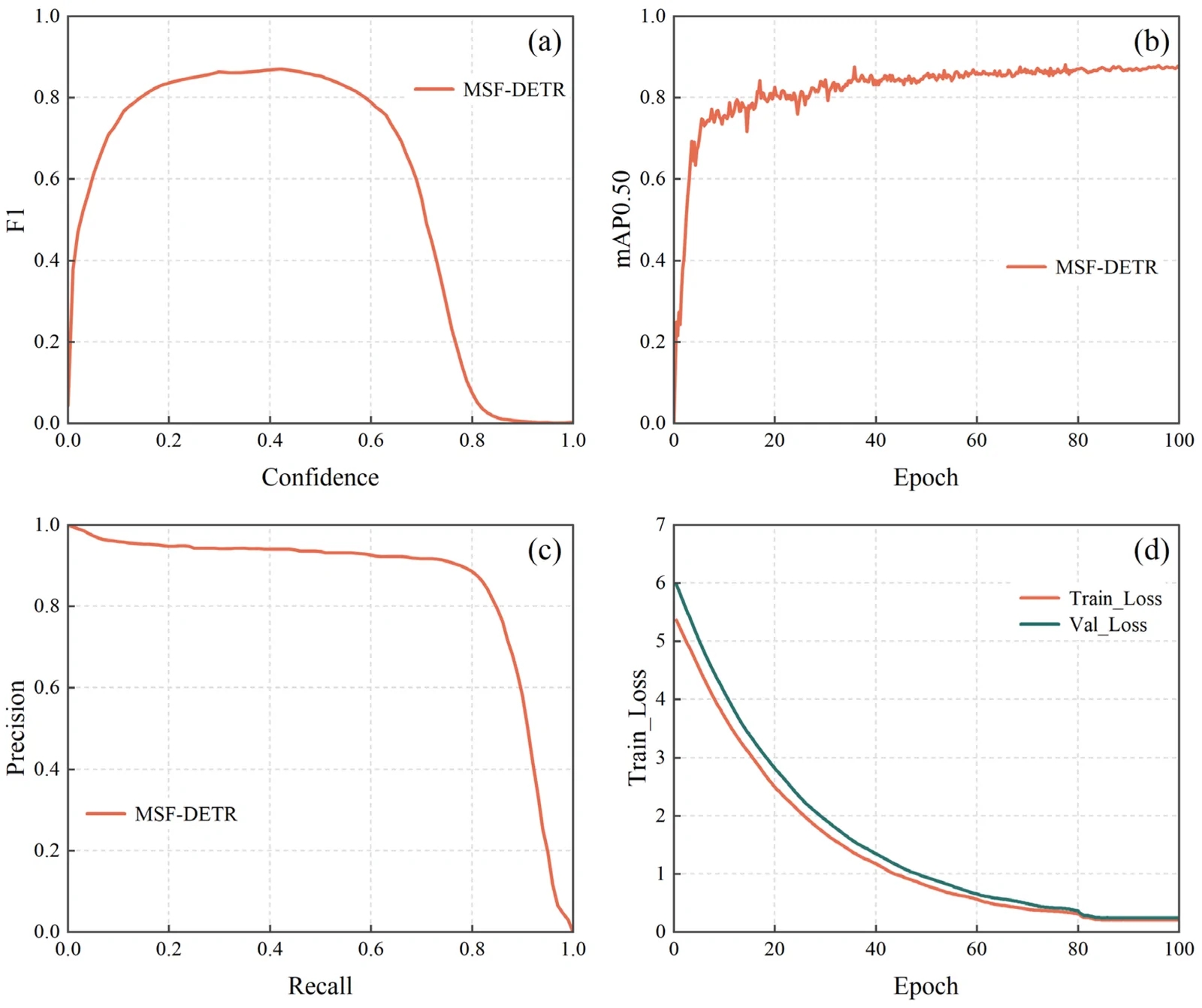
Training process curves of MSF-DETR. (**a**) F1 versus confidence, (**b**) mAP0.5 versus epoch, (**c**) precision–recall curve, and (**d**) training and validation loss curves.

To further evaluate the convergence behavior and training stability of MSF-DETR, the training process is visualized in Figure 7. Figure 7a shows the variation in the F1 score with confidence, where the model achieves its highest F1 within an intermediate confidence range, indicating a favorable balance between precision and recall. Figure 7b presents the evolution of mAP@0.5 over training epochs. The curve increases rapidly during the early training stage and gradually stabilizes after approximately 40 epochs, demonstrating rapid convergence and stable detection performance. Figure 7c illustrates the precision-recall curve, where MSF-DETR maintains relatively high precision across a broad recall range, confirming its robust detection capability under different confidence thresholds. Figure 7d shows the training and validation loss curves. Both losses decrease smoothly and converge to low values without significant divergence, indicating stable optimization and no obvious overfitting. Overall, the curves demonstrate that MSF-DETR achieves reliable convergence while maintaining stable detection performance throughout training.

Overall, the ablation experiments on both DIOR and DOTA consistently demonstrate the effectiveness and complementarity of the proposed components. EFC enhances cross-scale semantic interaction, FCA improves channel-wise feature discrimination, RAFM strengthens the preservation of fine-grained spatial information, and UQSL improves query-level supervision. Although the magnitude of improvement varies across individual configurations and datasets, the complete framework consistently achieves the highest mean values of APs, APm, APl, mAP50, and mAP50–95 on both datasets. In particular, the substantial improvements in APs on both DIOR and DOTA demonstrate the effectiveness of the proposed framework for small-object detection. Furthermore, the relatively small standard deviations obtained from 10 independent training runs indicate that the observed performance gains are robust to variations in random initialization. Meanwhile, the complete model introduces only a moderate increase in computational complexity while providing consistent improvements in detection accuracy. These results confirm that the proposed components are complementary and jointly contribute to a robust, stable, and effective aerial object detection framework.

### 4.5. Qualitative Visualization Analysis

**Figure 8:**
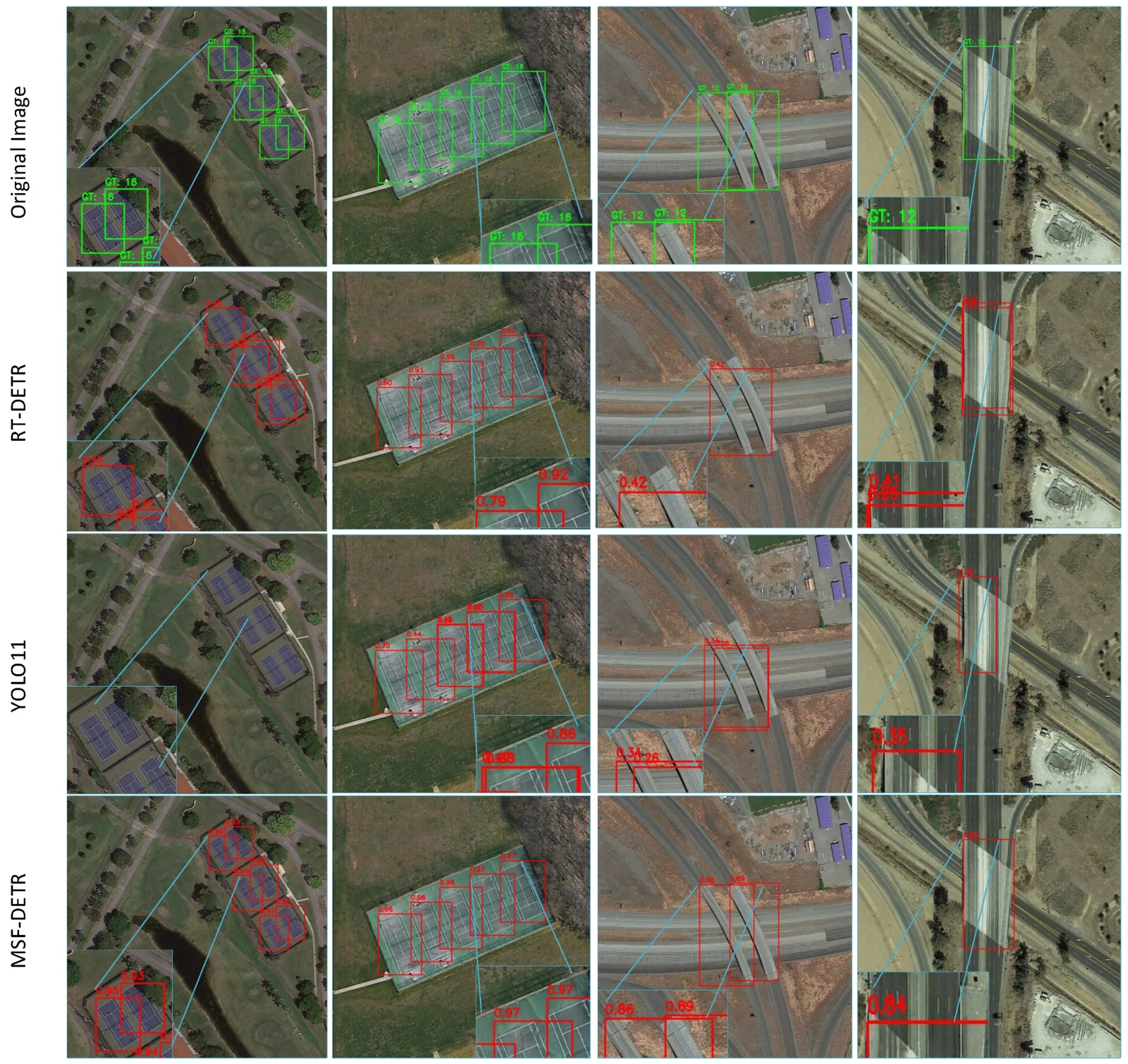
Comparative visualization of detection results for different algorithms.

To further assess the effectiveness of the proposed MSF-DETR, qualitative comparisons are conducted with representative object detectors, including RT-DETR and YOLO11. As shown in Figure 8, detection results are presented across several challenging UAV remote sensing scenarios characterized by dense object distributions, complex backgrounds, object scale variations, and partial occlusions. The compared scenes include densely arranged objects, road intersections, and objects with relatively small visual footprints, providing a comprehensive evaluation of the localization and recognition capabilities of different detectors.

In densely distributed scenes, such as the first and second columns of Figure 8, multiple objects are located in close proximity to each other, resulting in substantial spatial overlap and increased localization difficulty. RT-DETR is able to identify most of the objects but produces relatively coarse and inconsistent bounding boxes for several instances. YOLO11 also detects a considerable number of targets; however, some objects are missed, particularly in regions containing densely arranged instances. In comparison, MSF-DETR provides more complete detections and generates bounding boxes that are more closely aligned with the ground-truth annotations. These results indicate that MSF-DETR has a stronger capability to distinguish adjacent objects and maintain reliable localization in densely populated scenes.

The third column illustrates a more challenging road-intersection scenario, where elongated objects are distributed across regions with substantial background variation. In this case, both RT-DETR and YOLO11 exhibit relatively unstable localization performance, with some predicted bounding boxes deviating from the actual object boundaries or failing to completely cover the target. MSF-DETR produces more accurate and consistent bounding boxes, with improved alignment to the annotated object regions. This observation suggests that the proposed framework is better able to preserve discriminative spatial information under complex geometric layouts.

Small and distant objects present another important challenge in UAV-based object detection. As shown in the first, second, and fourth columns, some target instances occupy only a small portion of the image and contain limited discriminative visual information. YOLO11 shows reduced detection completeness in several of these regions, while RT-DETR improves the number of detected instances but still exhibits localization deviations for some small targets. In contrast, MSF-DETR successfully detects more small-scale objects and generally provides tighter bounding boxes. This demonstrates the enhanced sensitivity of the proposed detector to fine-grained target features and scale variations.

The fourth column further demonstrates the localization differences among the compared methods for elongated targets. Although both RT-DETR and YOLO11 are capable of identifying the major object instances, their predicted bounding boxes exhibit noticeable deviations in some cases. MSF-DETR achieves predictions that are more consistently aligned with the actual target extents, indicating improved localization robustness for objects with relatively large aspect-ratio variations.

Overall, the qualitative results demonstrate that MSF-DETR achieves more complete detections and more accurate bounding-box localization across diverse UAV remote sensing scenes. Compared with RT-DETR and YOLO11, the proposed method exhibits improved robustness in densely distributed, small-scale, and geometrically complex scenarios. These visual observations are consistent with the quantitative experimental results and provide further evidence that the proposed multi-scale feature representation and feature interaction strategy effectively enhance the model’s ability to capture discriminative information while maintaining accurate object localization.

**Figure 9:**
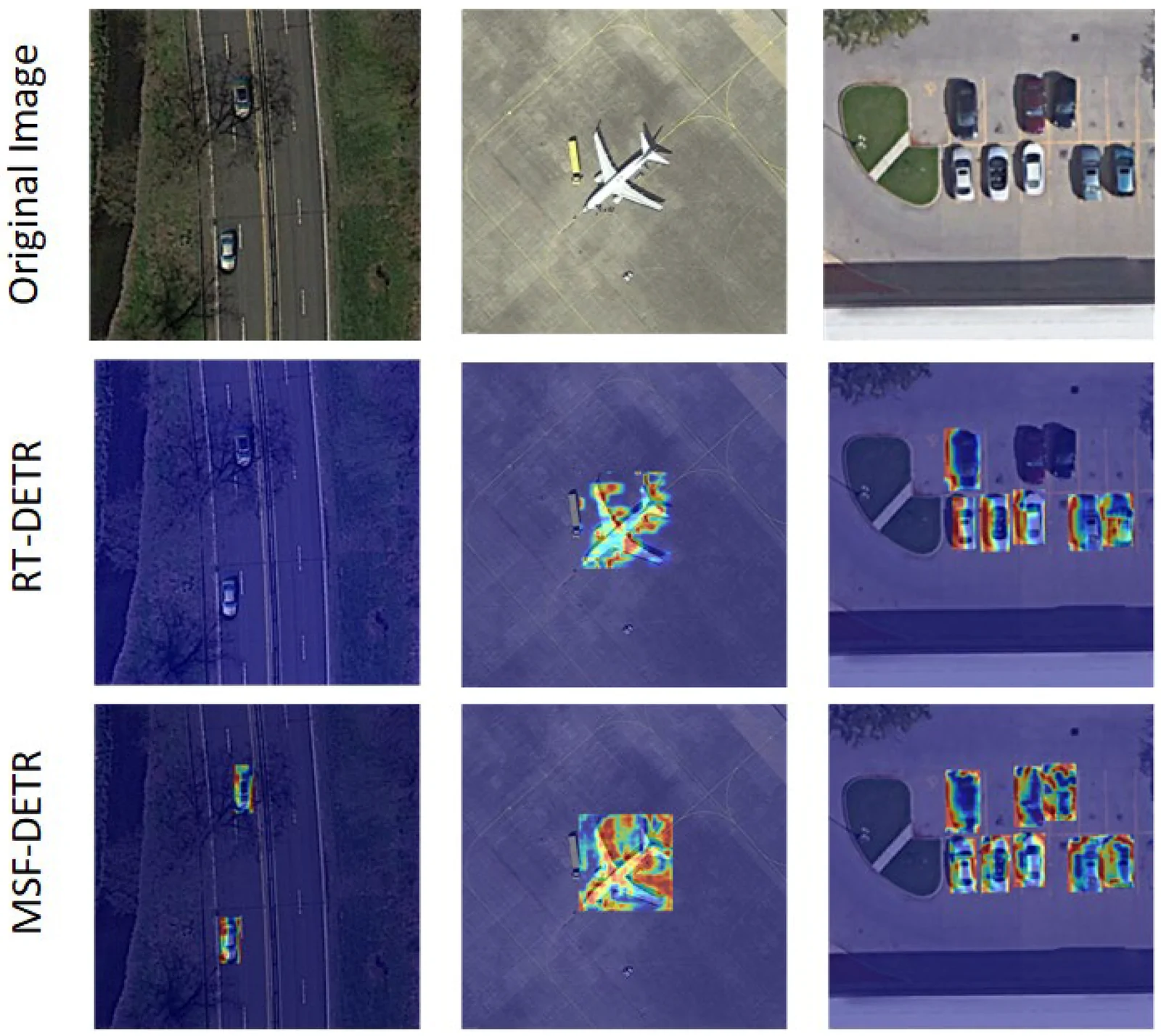
Visualization of attention heatmaps. The color scale represents the attention response intensity, with warmer colors indicating stronger responses and cooler colors indicating weaker responses.

To further investigate the feature representation and spatial attention characteristics of the proposed method, attention heatmaps are visualized to compare the responses of RT-DETR and MSF-DETR under different UAV remote sensing scenarios. As shown in Figure 9, three representative scenes containing road vehicles, an aircraft, and densely distributed vehicles are selected for qualitative comparison.

It can be observed that RT-DETR produces relatively scattered and fragmented activation patterns in several challenging regions. In the road vehicle scene, the responses of RT-DETR are relatively weak for some small vehicles, while the activation is not always consistently concentrated on the complete target regions. In the aircraft scene, RT-DETR generates strong responses around the aircraft, but the activation pattern is relatively irregular and fragmented, with responses distributed across different parts of the target. A similar phenomenon can be observed in the densely distributed vehicle scene, where the attention responses of RT-DETR vary considerably among individual vehicles.

In comparison, MSF-DETR generates more target-oriented and structurally coherent activation patterns. For the road vehicle scene, the proposed model produces clearer responses around both vehicles, including relatively small targets that receive weaker responses from RT-DETR. This indicates that MSF-DETR is capable of preserving and exploiting fine-grained features that are beneficial for small-object representation. For the aircraft scene, the high-response regions of MSF-DETR are more consistently distributed over the aircraft body and its main structural regions, resulting in a more complete representation of the target. In the densely distributed vehicle scene, MSF-DETR maintains relatively clear activation for multiple individual vehicles, demonstrating a stronger ability to distinguish neighboring targets in complex spatial arrangements.

Another noticeable difference is the response to background regions. Although both models generate certain activation outside the target areas, MSF-DETR generally maintains stronger responses within object regions while avoiding excessive activation over large irrelevant background areas. This suggests that the proposed feature enhancement and channel-selection mechanisms can help improve the discriminative quality of feature representations and reduce the influence of irrelevant information. In particular, the enhanced multi-scale feature interaction provided by the EFC module is expected to facilitate the preservation of weak and fine-grained features, while the FCA module can contribute to more selective feature responses by emphasizing informative channels.

Furthermore, the activation patterns produced by MSF-DETR exhibit relatively better spatial coverage of the target regions. Instead of being concentrated only on a few highly salient points, the responses tend to cover a larger portion of the object, particularly in the aircraft and densely distributed vehicle scenes. This behavior is consistent with the intended role of the RAFM module in enhancing spatial information propagation and maintaining fine-grained spatial representations. Meanwhile, the more consistent response patterns observed across different scenes are also compatible with the objective of UQSL, which is designed to improve the reliability and stability of query learning. However, these module-level interpretations should be considered complementary qualitative evidence, rather than direct causal proof from the heatmaps alone.

Overall, the visualization results demonstrate that MSF-DETR produces more target-oriented, complete, and discriminative attention patterns than RT-DETR across different UAV remote sensing scenarios. The proposed model shows particular advantages in small-object representation, densely distributed target discrimination, and suppression of irrelevant background responses. These qualitative observations are consistent with the quantitative improvements reported in the preceding experiments and provide an intuitive explanation for the enhanced detection performance of MSF-DETR in challenging UAV scenarios.

## 5. Conclusions

This paper presents MSF-DETR, a multi-scale feature enhancement detection framework for UAV aerial image object detection. Built upon RT-DETR, the proposed method improves feature representation and detection robustness under complex scenarios. Specifically, an Enhanced Feature Connection module is introduced to strengthen cross-scale interaction, while a Feature Channel Attention module improves discriminative capability by suppressing background interference. In addition, a Reinforced Attention Feedback Module preserves fine-grained spatial information within the Transformer encoder, and a Unified Query Supervision Loss stabilizes training and improves prediction consistency.

Experiments on the DIOR and DOTA datasets demonstrate that the proposed method achieves consistent improvements in detection accuracy and robustness, particularly in dense and small-object scenarios. Although the model introduces additional computational cost, it maintains a favorable balance between performance and efficiency. Future work will focus on lightweight design and extending the framework to more diverse UAV applications. Despite its improved performance, MSF-DETR still incurs additional computational cost and remains challenged by extremely small, occluded, and weak-response targets in complex backgrounds. Moreover, its robustness on diverse real-world UAV platforms and imaging conditions requires further validation. Future work will focus on lightweight and dynamic computation, improved scale-aware representation, model compression, hardware-aware optimization, and broader real-world evaluation.

## Figures and Tables

**Table 1:** Quantitative analysis of different weighting configurations for the unified query supervision loss (UQSL).

Configuration	$\lambda_{1}$	$\lambda_{2}$	$\lambda_{3}$	mAP50_50_ (%)	mAP50–95_50−95_ (%)
W1 (Equal)	1	1	1	86.34 ± 0.31	64.46 ± 0.27
W2	2	1	1	86.17 ± 0.33	64.31 ± 0.29
W3	1	2	1	86.12 ± 0.35	64.23 ± 0.30
W4	1	1	2	86.20 ± 0.30	64.29 ± 0.28
W5	3	1	1	86.02 ± 0.36	64.08 ± 0.31
W6	1	3	1	85.95 ± 0.38	63.94 ± 0.32
W7	1	1	3	86.00 ± 0.34	64.04 ± 0.30

**Table 2:** Component-level comparison between MSF-DETR and closely related methods.

Method	Cross-Scale Feature Fusion	Frequency-Domain Modeling	Attention Feedback	Denoising Supervision	One-to-Many Supervision	Query Optimization Mechanism
BiFPN	✓ Weighted bidirectional fusion	✗	✗	✗	✗	✗
FcaNet	✗	✓ Frequency-based channel attention	✗	✗	✗	✗
DN-DETR	✗	✗	✗	✓ Denoising queries	✗	✓ Hungarian matching
Group-DETR	✗	✗	✗	✗	✓ Group-wise assignment	✓ Group-wise query supervision
Co-DETR	✓ Auxiliary feature learning	✗	✗	✗	✓	✓ Collaborative hybrid assignment
MSF-DETR	✓ EFC: adaptive cross-scale reconstruction	✓ FCA: frequency-aware channel refinement	✓ RAFM: intermediate attention feedback	✓ UQSL	✓ UQSL	✓ Unified query supervision

Note: ✓ indicates that the corresponding feature or mechanism is included, whereas ✗ indicates that it is not included.

**Table 3:** Performance comparison summary table of MSF-DETR with other detectors on the DIOR dataset.

Model	Backbone	Params (M)	GFLOPs	FPS	mAP50 (%)	mAP50–95 (%)
Faster R-CNN [16]	ResNet50	58.0	99.2	18	62.3	45.7
DETR [17]	ResNet50	41.3	124	114	60.5	44.1
YOLOv5l [18]	CSPDarknet53	15.3	45.6	68	67.8	49.2
YOLOv7-Tiny [19]	CSPDarknet	6.2	13.7	62	61.4	45.3
YOLOv8n [20]	CSPDarknet53	3.01	8.1	65	64.6	47.5
YOLOv8s [21]	CSPDarknet53	11.2	28.5	70	69.5	51.8
YOLOv9s [22]	CSPDarknet53	5.0	14.0	70	70.1	52.6
YOLOv10s [23]	CSPDarknet53	5.6	15.2	74	73.8	53.3
YOLOv11s [24]	CSPDarknet53	6.0	16.8	74	75.6	57.2
LGFF-YOLO [25]	CSPDarknet	4.2	12.4	79.1	78.3	58.5
RFAG-YOLO [26]	CSPDarknet	5.9	15.7	82	79.6	54.9
DASSF [27]	CSPDarknet	8.5	23.9	46.7	76.5	56.8
YOLO-GE [28]	CSPDarknet	3.5	15.9	98.8	79.9	59.1
SSE-YOLO [29]	CSPDarknet	3.6	10.9	82.8	79.2	54.4
YOLO-MARS [30]	CSPDarknet	2.9	13.7	75.5	78.4	56.5
DAB-DETR [31]	ResNet50	44.1	94.0	38	82.7	60.4
Group-DETR [32]	ResNet50	45.8	98.3	34	84.1	62.2
Co-DETR [33]	ResNet50	52.7	118.4	26	85.2	63.1
RT-DETR [34]	ResNet50	42.5	85.1	81.3	83.5	61.8
MSF-DETR	ResNet50	44.6	91.4	73	86.3	64.4

**Table 4:** Performance comparison summary table of MSF-DETR with other detectors on the DOTA dataset.

Model	Backbone	Params (M)	GFLOPs	FPS	mAP50 (%)	mAP50–95 (%)
Faster R-CNN	ResNet50	58.0	99.2	18	68.3	41.2
DETR	ResNet50	41.3	124	114	66.7	39.8
YOLOv5l	CSPDarkNet53	15.3	46.5	68	72.1	44.5
YOLOv7	CSPDarkNet53	37.2	105.1	58	73.5	45.8
YOLOv8n	CSPDarkNet53	3.0	8.1	65	67.8	39.5
YOLOv8s	CSPDarkNet53	11.1	28.4	70	72.5	44.2
SMA-YOLO	CSPDarknet	2.6	4.5	73	70.3	42.8
FFCA-YOLO	CSPDarknet	7.1	51.2	56	69.1	42.1
FBRT-YOLO	CSPDarknet	2.9	22.9	143	67.5	40.6
DAB-DETR	ResNet50	44.1	94.0	38	74.6	46.3
Group-DETR	ResNet50	45.8	98.3	34	75.3	47.1
Co-DETR	ResNet50	52.7	118.4	26	76.1	47.8
RT-DETR	ResNet50	42.5	85.1	81.3	72.5	44.2
MSF-DETR	ResNet50	44.6	91.4	73	77.2	48.8

**Table 5:** The results of the ablation experiments on the DIOR dataset.

EFC	FCA	RAFM	UQSL	APs (%)	APm (%)	APl (%)	mAP50 (%)	mAP50–95 (%)	GFLOPs	Params (M)
–	–	–	–	31.27 ± 0.48	60.71 ± 0.42	72.84 ± 0.46	83.42 ± 0.37	61.76 ± 0.29	85.1	42.5
√	–	–	–	32.73 ± 0.45	61.94 ± 0.47	73.92 ± 0.51	84.16 ± 0.32	63.06 ± 0.36	86.6	43.0
–	√	–	–	33.64 ± 0.51	61.43 ± 0.39	73.62 ± 0.43	84.63 ± 0.41	62.58 ± 0.31	90.7	42.9
–	–	√	–	34.14 ± 0.43	62.16 ± 0.44	74.15 ± 0.48	84.86 ± 0.34	63.27 ± 0.32	88.4	44.1
–	–	–	√	32.57 ± 0.49	62.46 ± 0.41	74.91 ± 0.45	84.08 ± 0.38	63.58 ± 0.35	85.1	42.5
√	√	–	–	34.93 ± 0.46	62.76 ± 0.43	74.92 ± 0.47	85.35 ± 0.35	63.38 ± 0.30	91.6	43.3
√	√	√	–	35.94 ± 0.50	63.43 ± 0.45	75.46 ± 0.49	85.63 ± 0.33	64.07 ± 0.28	88.9	44.4
√	√	√	√	**38.06 ± 0.47**	**64.17 ± 0.40**	**76.94± 0.52**	**86.34 ± 0.31**	**64.46 ± 0.27**	91.4	44.6

Note: √ indicates that the corresponding module is included in the model, whereas – indicates that the module is not included. Bold values indicate the best-performing architecture variant among the compared configurations.

**Table 6:** The results of the ablation experiments on the DOTA dataset.

EFC	FCA	RAFM	UQSL	APs (%)	APm (%)	APl (%)	mAP50 (%)	mAP50–95 (%)	GFLOPs	Params (M)
–	–	–	–	20.43 ± 0.59	43.31 ± 0.56	59.18 ± 0.53	72.43 ± 0.47	44.14 ± 0.39	85.1	42.5
√	–	–	–	21.94 ± 0.55	44.52 ± 0.51	60.31 ± 0.57	73.76 ± 0.43	45.24 ± 0.41	86.6	43.0
–	√	–	–	21.46 ± 0.52	43.91 ± 0.57	59.84 ± 0.50	73.18 ± 0.46	44.87 ± 0.38	90.7	42.9
–	–	√	–	22.26 ± 0.58	44.42 ± 0.53	60.21 ± 0.55	73.59 ± 0.42	45.06 ± 0.37	88.4	44.1
–	–	–	√	21.73 ± 0.54	44.87 ± 0.50	60.94 ± 0.52	74.03 ± 0.45	45.65 ± 0.40	85.1	42.5
√	√	–	–	23.14 ± 0.56	45.61 ± 0.54	61.43 ± 0.58	74.68 ± 0.39	46.27 ± 0.34	91.6	43.3
√	√	√	–	24.03 ± 0.61	46.02 ± 0.52	61.91 ± 0.56	75.08 ± 0.41	46.57 ± 0.36	88.9	44.4
√	√	√	√	**26.14 ± 0.57**	**48.32 ± 0.60**	**64.71 ± 0.63**	**77.16 ± 0.38**	**48.74 ± 0.35**	91.4	44.6

Note: √ indicates that the corresponding module is included in the model, whereas – indicates that the corresponding module is not included. Bold values indicate the best-performing results among the compared configurations.

## Data Availability

The data presented in this study are available upon reasonable request from the corresponding author. The data are not publicly available due to privacy restrictions.

## Abbreviations

UAV	Unmanned Aerial Vehicle
MSF-DETR	Multi-Scale Feature Enhancement Detection Transformer
RT-DETR	Real-Time Detection Transformer
DETR	Detection Transformer
CNN	Convolutional Neural Network
EFC	Enhanced Feature Connection
FCA	Feature Channel Attention
RAFM	Reinforced Attention Feedback Module
UQSL	Unified Query Supervision Loss
FPN	Feature Pyramid Network
GFF	Gated Feature Fusion
MFR	Multi-scale Feature Reconstruction
DA	Deformable Attention
FFN	Feed-Forward Network
DN-DETR	Denoising Detection Transformer
FPS	Frames Per Second
GFLOPs	Giga Floating-point Operations Per Second
mAP	Mean Average Precision
mAP50	Mean Average Precision at IoU threshold 0.50
mAP50–95	Mean Average Precision averaged over IoU thresholds from 0.50 to 0.95
DIOR	Dataset for Object Detection in Optical Remote Sensing Images
DOTA	Dataset for Object Detection in Aerial Images
IoU	Intersection over Union
RPN	Region Proposal Network
NMS	Non-Maximum Suppression
LCL	Linear-Convolution-Linear
GIoU	Generalized Intersection over Union
SNR	Signal-to-Noise Ratio
